# Timescales of the permafrost carbon cycle and legacy effects of temperature overshoot scenarios

**DOI:** 10.1038/s41467-021-23010-5

**Published:** 2021-05-11

**Authors:** Philipp de Vrese, Victor Brovkin

**Affiliations:** 1 Max Planck Institute for Meteorology, The Land in the Earth System, Hamburg, Germany; 2 Center for Earth System Research and Sustainability, University of Hamburg, Hamburg, Germany

**Keywords:** Climate and Earth system modelling, Cryospheric science

## Abstract

Minimizing the risks and impacts of climate change requires limiting the global temperature increase to 1.5 °C above preindustrial levels, while the difficulty of reducing carbon emissions at the necessary rate increases the likelihood of temporarily overshooting this climate target. Using simulations with the land surface model JSBACH, we show that it takes high-latitude ecosystems and the state of permafrost-affected soils several centuries to adjust to the atmospheric conditions that arise at the 1.5 °C-target. Here, a temporary warming of the Arctic entails important legacy effects and we show that feedbacks between water-, energy- and carbon cycles allow for multiple steady-states in permafrost regions, which differ with respect to the physical state of the soil, the soil carbon concentrations and the terrestrial carbon uptake and -release. The steady-states depend on the soil organic matter content at the point of climate stabilization, which is significantly affected by an overshoot-induced soil carbon loss.

## Abstract (web summary)

In this study, the authors investigate a scenario where global temperature increase is limited to 1.5 °C. They find that Arctic ecosystems will need centuries to adapt to such an increase and that the ensuing steady-state depends on the preceding climate trajectory.

## Introduction

During the last glacial cycle, soils in the high northern latitudes have accumulated vast organic carbon pools^1–5^. The largest fraction of these is located in regions underlain by permafrost where sub-zero temperatures have been protecting the organic material from degradation. Rising temperatures in the Arctic diminish the spatial extent and thickness of the permafrost, leaving more soil organic matter (SOM) vulnerable to decomposition^6–15^. This raises the soil’s CO_2_ and CH_4_ emissions which in turn contribute to the rise in temperatures^16–18^. With roughly 1100–1700 Gt of carbon stored in northern permafrost regions^19–21^ and Arctic temperatures increasing twice as fast as the global average^22^, this positive feedback is becoming increasingly important, giving scientists reason to consider permafrost to be one of the key tipping elements of the climate system^23–27^.

Numerous studies have focused on the thermal degradation of permafrost under different warming scenarios, including the resulting SOM loss and the associated release of greenhouse gases (GHG)^27–33^. However, the system’s long-term response to past and future climate change remains highly uncertain. In particular, it is unclear how fast the state of soils and the carbon fluxes will adapt to steady climate conditions in regions where low temperatures result in extremely long carbon turnover times^34,35^ and where the soil characteristics—especially a high water content—can lead to a large thermophysical inertia^36^. Furthermore, it is unknown whether the resulting steady-state depends on the climate pathway that precedes the stable period. The latter is especially important in the view of carbon emission pathways that are compatible with the Paris agreement’s long-term goal of maintaining the global mean temperature well below 2 °C above preindustrial levels. Since an abrupt reduction of emissions is likely to entail extreme economic costs, many future scenarios consider temporarily overshooting the climate target—so called temperature overshoot (OS) scenarios^37,38^. Here, the permissible CO_2_ emissions to meet or stay below these targets could even be lower than previously thought, partly because permafrost carbon feedbacks have not been fully taken into account^16–18^.

Frozen organic compounds are considered stable and from observations we know that permafrost can conserve the organic matter—at least—over tens of thousands of years^1–5^. Thus, the carbon concentrations below the active layer reflect the state of the ecosystem at the time the respective soil region was incorporated into the permafrost. This state is determined by the antecedent climate trajectory, making it possible that a temporary warming would affect the amount of organic matter stored within the perennially frozen fraction of the soil, changing the emission budget for a given temperature target^16–18^. However, it is an open question whether an OS could entail long-lasting legacy effects for the Arctic ecosystem that go beyond the amount of stable permafrost carbon, affecting soil temperatures, the soil water content or even primary productivity and soil respiration. Here, previous studies do not only indicate that the global carbon budgets of different OS scenarios would eventually converge^39^, but also that the equilibrium state of permafrost-affected soils is independent of the preceding climate trajectory, with any hysteretic behavior being merely transient^36^. These studies, however, did not account for the full range of feedbacks between physical and biophysical soil processes, neglecting the impact of SOM on the soil thermal properties and the resulting effect on soil carbon accumulation rates^40^.

**Fig. 1:**
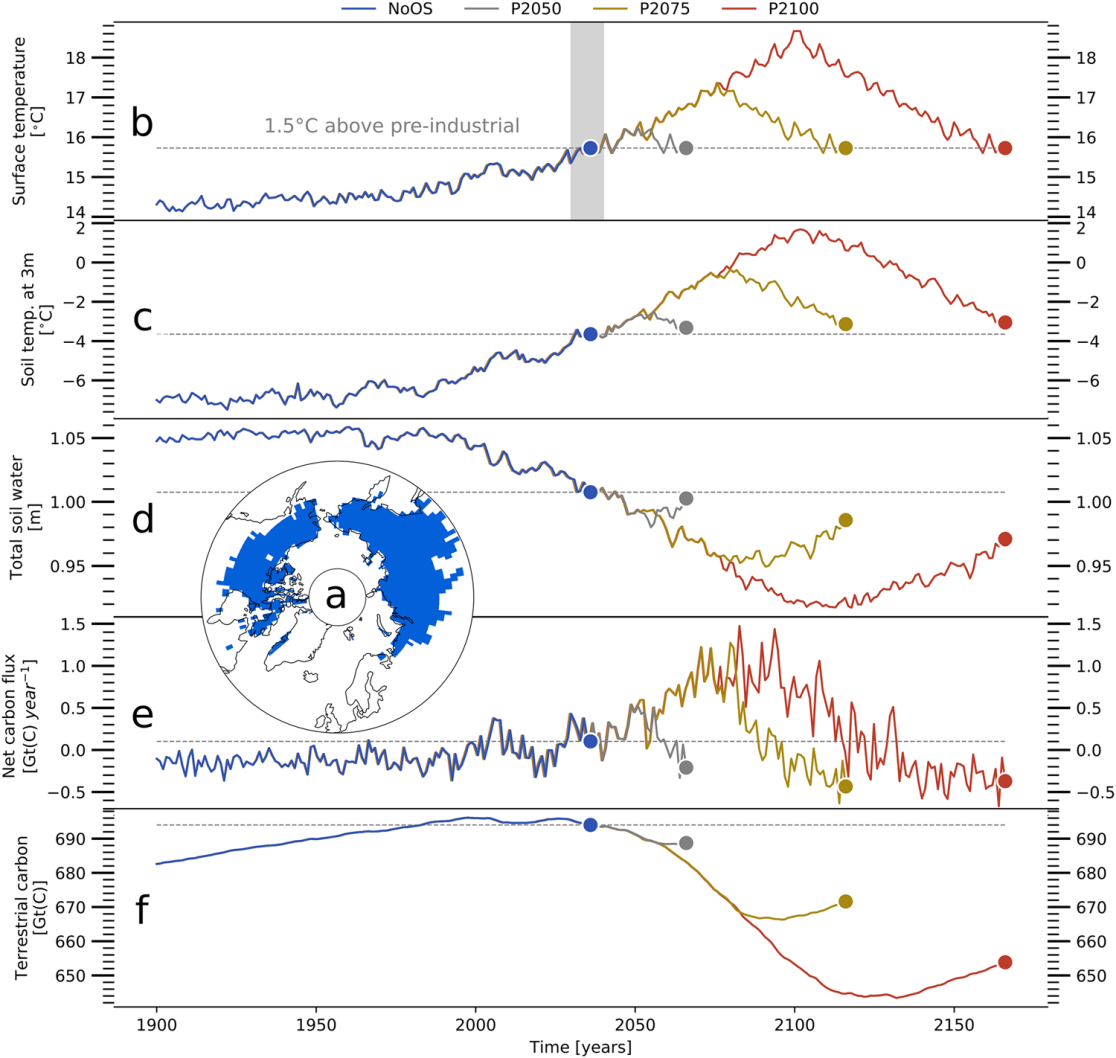
Pathways to the Paris agreement’s long-term temperature goal. **a** Regions in the high northern latitudes that are affected by near-surface permafrost: blue color shows non-glacier grid cells in which the simulations featured perennially frozen soils—above a depth of 3 m—at the beginning of the twenty-first century. Panels show four scenarios that reach the target climate (PACT1.5) after different temperature overshoots (OS)—**b** simulated (global mean) temperature, **c** annual mean soil temperature at a depth of 3 m, **d** total soil water (liquid and frozen water within the top- and subsoil which can reach to a depth of 10 m), **e** net ecosystem carbon emissions, **f** total terrestrial carbon (soil carbon and vegetation biomass). NoOS (blue lines) constitutes a scenario in which atmospheric greenhouse gas concentrations follow the high emission-trajectory according to SSP5-8.5^67,68^ until the year 2035 and the PACT1.5 is met without OS. P2050 (gray lines) depicts a scenario that follows SSP5-8.5 until the year 2050 before reversing to the PACT1.5, while P2075 (yellow lines) assumes a peak in 2075 and P2100 (red lines) a peak in 2100. **b** Indicates the global mean, while **c**–**e** give the averages over the northern permafrost regions—indicated in **a**. The gray shading in **b** indicates the period (2030–2040) during which the atmospheric conditions correspond to the PACT1.5.

In the following, we use simulations with JSBACH, the land surface component of the Max-Planck-Institute for Meteorology’s Earth system model MPI-ESM1.2^41^, to explore the timescale on which the Arctic ecosystem adjusts to the atmospheric conditions that result from climate stabilization at a Paris-Agreement-compliant temperature target (PACT1.5)—more specifically at 1.5 °C above preindustrial levels (Sec. 2, part 1). Here, we show that it takes high-latitude ecosystems and the state of permafrost-affected soils several centuries to adjust to the ensuing atmospheric conditions. Furthermore, we address the question whether a preceding OS could entail consequences for the Arctic carbon cycle and the state of permafrost-affected soils that are irreversible under the ensuing, nontransient atmospheric conditions (Sec. 2, part 2), showing that feedbacks between the water-, energy-, and carbon cycles, indeed, allow for multiple steady-states in permafrost regions. To this end we investigate simulations—with prescribed atmospheric conditions—that target the state of the northern permafrost regions (Fig. 1a) after reaching the PACT1.5 by different climate trajectories (Fig. 1b and Supplementary Fig. 5): without prior OS and after three different OS scenarios that are based on SSP5-8.5 and assume forcing-peaks in the years 2050, 2075, and 2100, respectively. It should be noted that the simulations in this study were not performed with the standard JSBACH model. A short overview over the adapted version is given in the methods section, while a more detailed description of the modifications to the model is provided in de Vrese et al.^42^ and a brief comparison between the model versions and to observational data is included in the supplements (Supplementary Figs. 1–3; note that the present JSBACH version is largely based on the work of Ekici et al.^43^ and their model version is included in the intercomparison). Furthermore, the following investigation focuses on the dynamics under nontransient conditions, while a detailed description of the dynamics during different OS scenarios can also be found in de Vrese et al.^42^.

## Results

### Adjustment timescales in permafrost regions

When reaching a given temperature target, the state of the soils in permafrost-affected regions initially depends on the preceding climate trajectory—in case of an OS, on the temperature-, precipitation-, and atmospheric GHG excess as well as on the OS’s duration. For the PACT1.5, the simulated soil temperatures—at a depth of 3 m—differ by 0.3–0.5 °C after climate stabilization, corresponding to 10–30 % of the temperature increase during the antecedent OSs (Fig. 1c and Table 1). The total water content of the soil (frozen and liquid) varies by up to 5 cm—or 50 l m^−2^—which is as much water as the soils loose during the historical period (Fig. 1d). In addition to the physical state of the soil, the climate trajectory also affects key aspects of the Arctic carbon cycle.

**Table 1:** Overview over key variables in the scenario simulations leading to the PACT1.5: Global mean surface temperature (*T*${}_{{{{{\rm{surf}}}}}^{+1}}$), soil temperature at a depth of 3 m (*T*_3 m_), permafrost volume within the top 3 m of the soil (Perm_top3_), total soil water (H_2_O_tot_), vegetated fraction (Veg${}_{\max }$), total terrestrial carbon stocks (*C*_tot_), total soil carbon (*C*_soil_), net primary productivity (NPP), soil respiration (*R*_soil_), and net ecosystem carbon flux into the atmosphere (NEF). Values refer to a given variable when reaching the PACT1.5 (20 year running mean), while values in brackets refer to the preceding peak. With the exception of T_surf_, all values constitute the average or accumulation over the permafrost-affected regions (Fig. 1a).

Simulation	*T*${}_{{{{{\rm{surf}}}}}^{+1}}$ (°C)	*T*_3m_ (°C)	Perm_top3_ (%)	H_2_O_tot_ (m)	Veg${}_{\max }$ (%)	*C*_tot_ (GtC)	*C*_soil_ (GtC)	NPP GtC (year^−1^)	*R*_soil_ GtC (year^−1^)	NEF GtC (year^−1^)
NoOS	15.7	−3.6	56	1.01	71	689	665	4.51	4.47	0.18
P2050	15.7 (16.2)	−3.3 (−2.7)	51 (48)	0.99 (1.00)	74 (74)	685 (687)	653 (657)	4.88 (5.28)	4.66 (5.35)	0.03 (0.34)
P2075	15.7 (17.4)	−3.1 (−0.8)	49 (26)	0.98 (0.96)	81 (81)	668 (671)	622 (631)	5.17 (7.22)	4.63 (7.61)	−0.26 (0.88)
P2100	15.7 (18.7)	−3.1 (1.6)	49 (9)	0.96 (0.93)	85 (86)	650 (649)	594 (595)	5.22 (9.0)	4.72 (9.2)	−0.24 (0.77)

^+1^ denotes global mean.

The terrestrial net carbon emissions indicate whether the high latitudes constitute a sink of atmospheric CO_2_ or a carbon-source that requires further reductions in the anthropogenic emissions in order to stabilize global temperatures at the target level. Under preindustrial and present climate conditions, the vegetation in the high latitudes is thought to take up more carbon from the atmosphere than is emitted by heterotrophic respiration and wildfires (note that the model does not include carbon leaching or the processes in aquatic systems)^44^. However, when reaching the PACT1.5—without OS—the permafrost-affected regions have turned into a source for atmospheric CO_2_ with net fluxes into the atmosphere of around 0.2 GtC year^−1^ (Fig. 1e). The positive emissions are partly a consequence of the carbon release from recent and ongoing permafrost degradation, resulting in particularly long adjustment timescales (see below). In contrast, the high latitudes constitute a carbon sink when the PACT1.5 is reached subsequent to an OS—with a net uptake of up to 0.3 GtC year^−1^. On the one hand, a given fraction of the formerly frozen SOM is decomposed during the OS, reducing the SOM content and soil respiration rates in the subsequent stable period. On the other hand, the vegetation has adapted to the warmer conditions and the increased atmospheric GHG concentrations of the OS period, which leads to a higher net primary productivity, higher carbon inputs into the soil and, consequently, higher soil respiration rates. Overall, the effects of the reduction in formerly frozen SOM outweigh those stemming from the increased substrate availability due to fresh carbon inputs, especially as the higher CO_2_ uptake by the vegetation does not only increase the litter flux but also the (living) vegetation biomass. As a result, the total terrestrial carbon is only up to 40 GtC lower after than before the OS (Fig. 1f), while the amount of SOM differs by up to 70 GtC (Table 1).

**Fig. 2:**
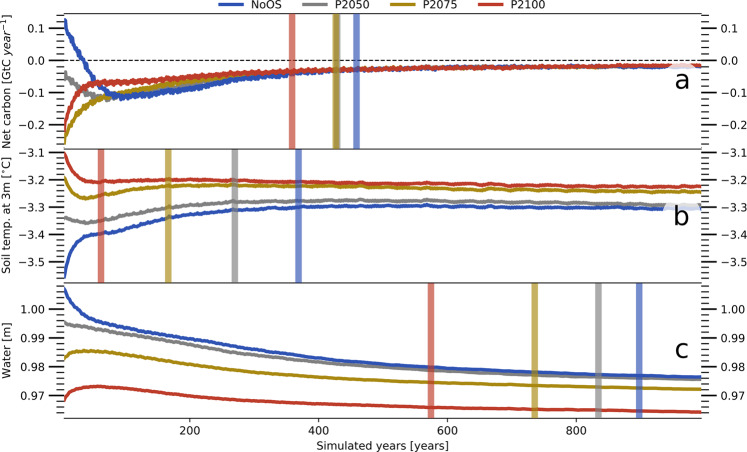
High-latitude inertia. Shown is the development of key variables under the nontransient atmospheric conditions that arise at the target climate (PACT1.5). **a** Simulated net ecosystem carbon fluxes into the atmosphere, **b** soil temperatures at a depth of 3 m, and **c** total (liquid and frozen) water content of the soil—in the permafrost-affected areas of the high northern latitudes. Blue lines refer to simulations (NoOS) without previous temperature overshoot (OS), gray lines indicate simulations that started subsequent to an OS with a peak in 2050 (P2050), yellow lines after an OS with a peak in 2075 (P2075) and red lines after an OS with a peak in 2100 (P2100). Shown is the 10-year running mean and solid vertical lines indicate the adjustment timescale. The latter considers the duration until a given variable has largely adapted to the atmospheric conditions—defined by the point at which the rate of change of a given variable has decreased by an order of magnitude (“Methods”). It should be noted that the analysis is based on the spatial average over the simulated permafrost regions, while individual grid cells can still exhibit substantial trends when the high latitudes as a whole have largely adjusted to the steady atmospheric conditions (see below).

The state upon reaching the temperature target, in turn, determines how the system behaves in the nontransient climate, in particular how fast the terrestrial carbon-, energy-, and water cycles adapt to the prevailing atmospheric conditions. In case of climate stabilization without prior OS, it takes the simulated high-latitude net carbon flux roughly 450 years to largely adjust to the atmospheric conditions (Fig. 2a). The first 100 years of this period are characterized by a marked decrease in the net emissions resulting from the rising CO_2_ uptake by the expanding vegetation, which increasingly outweighs the emissions from the decomposition of formerly frozen SOM. After the vegetation has largely adapted to the prevailing atmospheric conditions, litter and SOM start to accumulate, raising the soil carbon emissions. In the following centuries, this increases the ecosystem net fluxes substantially. However, the continental Arctic remains a carbon sink under PACT1.5 conditions, at least until the end of the first millennium following climate stabilization. The ecosystem flux behaves very different when the PACT1.5 is reached subsequent to an OS. Here, the dynamics depend on the extent to which the vegetation has adapted to the more favorable conditions during the OS, including the build up of fresh litter and SOM, but also on the amount of formerly frozen SOM that has been respired. Overall, the adjustment period is notably shorter and for a large OS—following SSP5-8.5 until the year 2100—the net fluxes adjust about 100 years earlier.

In Earth system models, the vegetation dynamics and the decomposition of SOM have to be parameterized^45,46^, making the exact adjustment timescale highly dependant upon the assumptions that go into any particular model. Nonetheless, the order of magnitude of the timescale—that is centuries rather than decades—and the fact that there are still minor trends even after 1000 years under PACT1.5 conditions, clearly indicate that within our lifetime we will not see the high-latitude ecosystem fully adjust to the atmospheric conditions that result from meeting the PACT1.5. Furthermore, the timescale on which the net carbon fluxes adapt is also determined by the physical inertia of the permafrost-affected regions. The vegetation’s primary productivity and the decomposition rates of SOM depend on the below ground temperatures and the availability of liquid water, linking both the carbon uptake and release to the physical state of soil.

Soils have a large heat capacity, while the conductive transport—heat transmission from particle to particle—is comparatively slow, delaying any temperature signal with increasing depth. But the main factor determining the thermal inertia is the energy required for or released in the phase change of water. Here, the system is particularly inert in the case of increasing temperatures, when a substantial amount of energy is needed to melt the ice within the soil. Consequently, it takes almost 400 years until the soil temperatures have largely adapted to PACT1.5 conditions, if these are reached without preceding OS (Fig. 2b). In contrast the soil temperatures adjust within the first century if the PACT1.5 is reached after a large OS. However, independent of the preceding climate trajectory the soils exhibit a minor cooling trend even after 1000 years under nontransient atmospheric conditions.

The temperatures in turn have an important impact on the hydrological conditions in the soil. Sub-zero temperatures immobilize the soil water, inhibiting it’s vertical transport through the ground. Thus, permafrost essentially acts a boundary that impedes drainage and when the permafrost degrades in response to rising temperatures, this boundary disappears and the soils desiccate (Fig. 2c). The drying process is extremely slow because it depends not only on the near-surface permafrost but also on the soil temperatures at greater depths. Additionally, small precipitation-evapotranspiration (P-E) deficits can reduce the soil water content over long periods before the drying affects the plant available water to a degree that decreases transpiration rates, eventually balancing the P-E deficits. Thus, even after an extreme OS it takes several centuries for the simulated soil water contents to adapt to the PACT1.5 conditions and even then a minor drying trend remains.

**Fig. 3:**
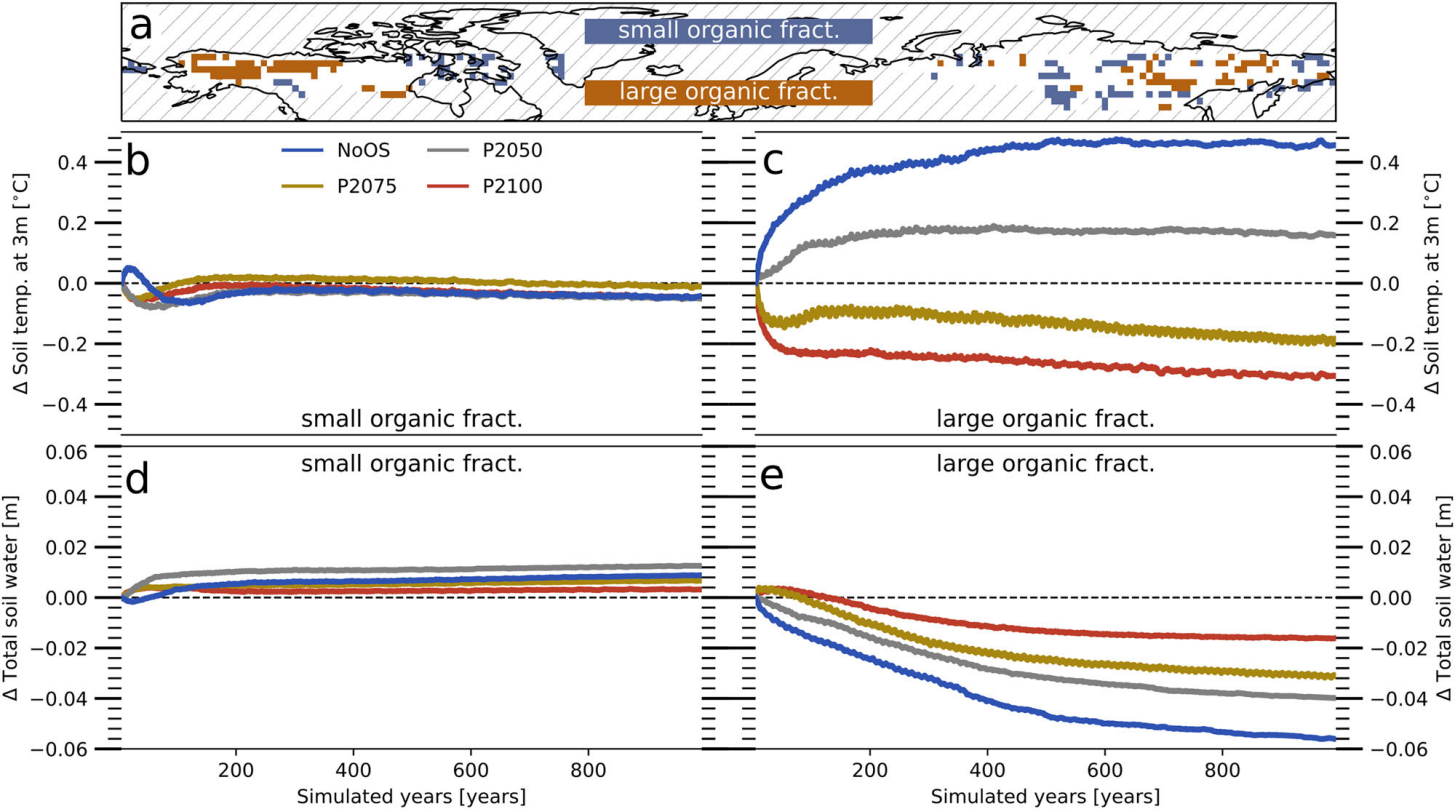
Different dynamics in largely mineral and largely organic soils. **a** Grid cells that retain largely mineral (brown) or organic (blue) soils under the nontransient atmospheric conditions that arise at the target climate (PACT1.5). Here, largely mineral soils are simply defined as those containing <25 kg(C) m^−2^, while predominantly organic soils are defined by an organic matter content >75 kg(C) m^−2^. **b** Temperature development in grid cells that retain largely mineral soils. Temperatures are given relative to the value at stabilization. Blue lines refer to simulations (NoOS) without previous temperature overshoot (OS), gray lines indicate simulations that started subsequent to an OS with a peak in 2050 (P2050), yellow lines after an OS with a peak in 2075 (P2075), and red lines after an OS with a peak in 2100 (P2100) . **c** Same as **b**, but for organic soils. **d** Same as **b**, but showing the total water content of the soil. **e** Same as **c**, but showing the total water content of the soil.

With respect to the water- and energy cycle, the adjustment timescales are highly dependent on the properties of the soil, especially on the soil porosity^36^. The latter largely determines the amount of water—liquid and frozen—the soil can hold and it is highly relevant for heat transport as air has a comparatively low heat conductivity. The soil properties are partly determined by the SOM, which has a particularly high porosity. Thus, while the continental Arctic as a whole takes centuries to adapt to the PACT1.5 conditions, larger trends in temperature and water content are only present in soils that have a high carbon content while regions with predominantly mineral soils quickly adapt to the prevailing atmospheric conditions (Fig. 3).

### Multistability in high-latitudes and legacy effects of overshoot scenarios

Our simulations do not only indicate very long adjustment timescales in the permafrost-affected regions, but also show notable differences between the scenarios even after 1000 years under the same atmospheric conditions (Fig. 2). If these differences resulted exclusively from the OS’s direct impact on the physical parameters of the permafrost ecosystem, the simulations would eventually converge to the same equilibrium state. Additional energy in the soil would be released at the surface in the form of larger turbulent heat- and outgoing long-wave radiative fluxes, while the soil water content would be adjusted by differing infiltration-, drainage-, and evapotranspiration rates. However, even after most parameters have largely adapted to the nontransient atmospheric conditions, there is a distinct offset between the scenarios, indicating interactions with the carbon cycle that could sustain differing states over a much longer period.

Higher temperatures increase the microbial activity and predominantly reduce the amount of carbon stored in high latitude soils (Table 1). The SOM, in turn, has a strong impact on the soil’s hydrological and thermal properties^40^ (an overview over the respective implementation in JSBACH is given in Supplementary Fig. 4). Consequently, any OS affects all physical and biophysical processes also indirectly by altering the boundary conditions under which these processes occur. And while the direct effects—the ones originating in the OS’s direct impact on the soil’s energy and water content—diminish over time, the feedbacks between physical and biophysical processes have the potential to stabilize the carbon-, water-, and energy cycles at different equilibria.

**Fig. 4:**
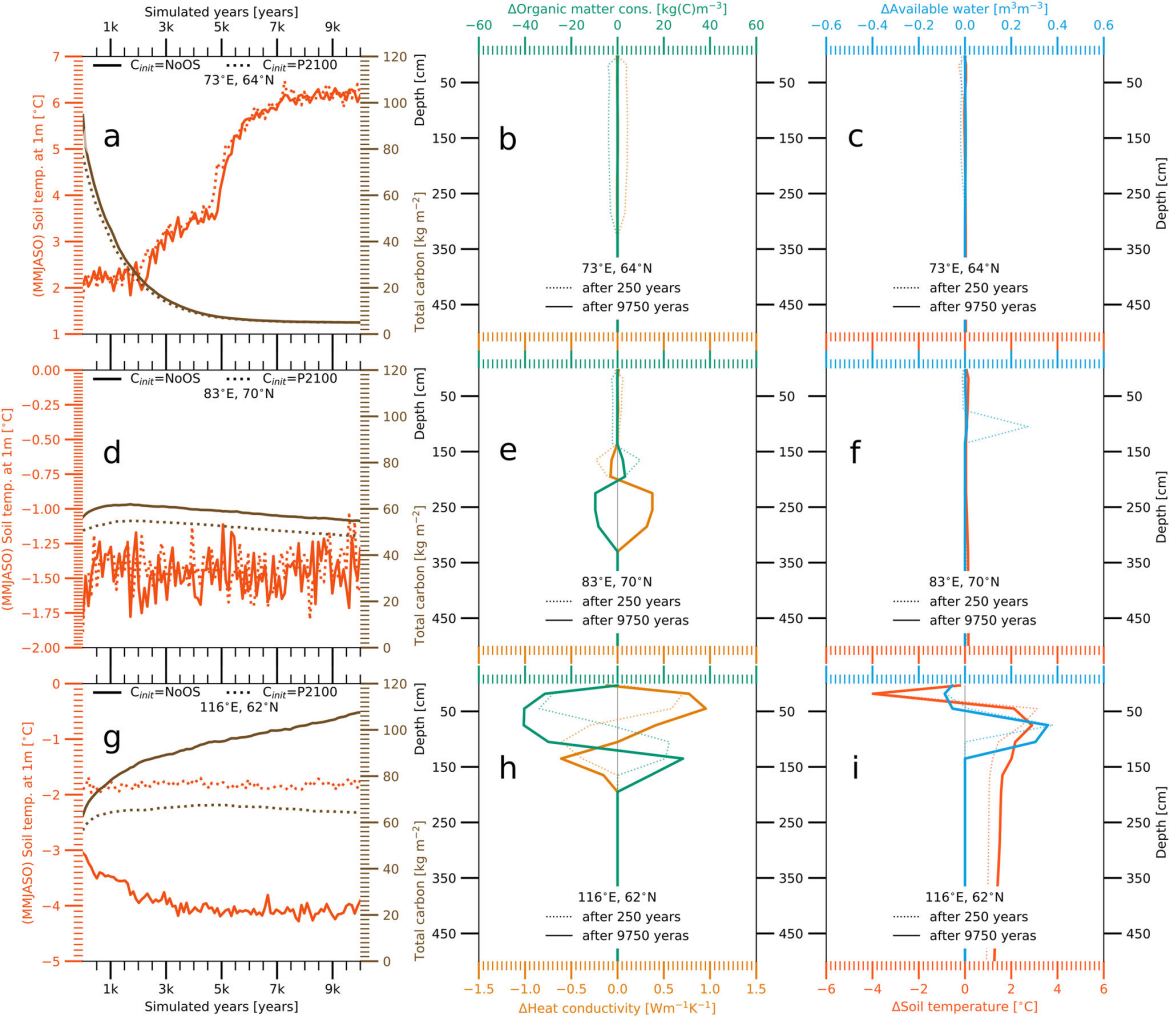
Dependency of steady-state conditions on the initial soil carbon concentrations. **a** Simulated May–October mean soil temperatures at a depth of 1 m (red lines, left *y*-axis) and carbon densities (brown lines, right *y*-axis) in a grid cell at 73°E; 64°N. Solid lines refer to a simulation which was initialized with soil carbon pools before the temperature overshoot (NoOS), whereas dotted lines show a simulation that started from the pools after the overshoot (P2100). Both simulations were initialized with the same soil temperature profile and soil water content and were similarly forced with prescribed atmospheric conditions corresponding to the target climate (PACT1.5) . All lines show 100-year averages. **b** Difference in soil organic matter concentration (green lines, top *x*-axis) and heat conductivity (yellow lines, bottom *x*-axis) between the two simulations (P2100-NoOS), at the beginning (dotted lines) and at the end (solid lines) of a 10,000-year period. **c** Same as **b** but for the annual maximum (monthly mean) soil temperature (red lines, top *x*-axis) and liquid water content (blue lines, bottom *x*-axis). **d**, **g** Same as **a** but for grid cells located at 83°E; 70°N and 116°E; 62°N. **e**, **h** Same as **b** but for grid cells located at 83°E; 70°N and 116°E; 62°N. **f**, **i** Same as **c** but for grid cells located at 83°E; 70°N and 116°E; 62°N. Please note that further examples are given in Supplementary Fig. 6.

In grid cells where the near-surface permafrost disappears, the soil carbon concentration, temperatures, and water content converge on the same state, independent of the initial conditions (Fig. 4a–c). However, this is not necessarily the case for grid cells that retain permafrost under PACT1.5 conditions. In a number of these grid boxes we find long-lasting differences in the SOM between simulations that were initiated with the carbon pools before and after an OS. In some cases these differences are limited to the carbon content within the frozen fraction of the ground and are a result of the higher temperatures during the OS. These temporarily increase the active layer thickness, allowing to decompose SOM that would otherwise have remained frozen (Fig. 4d–f). But more importantly, we often find persistent differences in the soil carbon concentration to coincide with sustained differences in the soil temperatures, especially those during the warmer period of the year—May—October (MJJASO). Here, cooler soils (below a depth of ≈0.5 m) can lead to a prolonged carbon accumulation, while warmer soils stabilize the SOM at lower concentrations. In extreme cases, the lower below ground temperatures entail a steady-state in which the soils continuously accumulate organic matter, while the warmer soils promote a steady SOM loss (Fig. 4g–i). Under these circumstances the resulting soil composition can differ substantially, with organic soils being simulated for higher initial soil carbon concentrations, whereas lower carbon pools upon climate stabilization result in a higher mineral fraction. This is a strong indicator that parts of the northern high latitudes are multi-stable and that the steady-state of permafrost soils is affected by the soil carbon loss due to a potential OS.

**Fig. 5:**
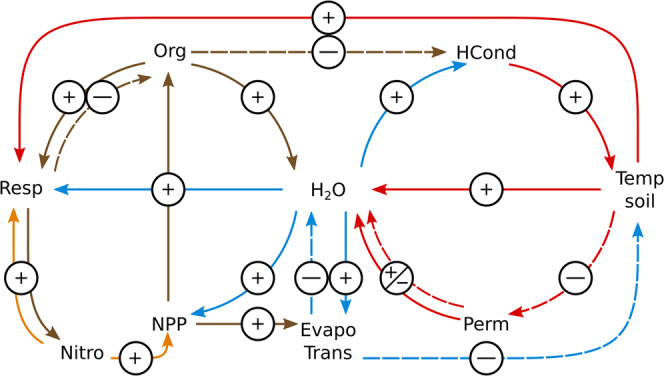
Feedbacks between soil carbon-, water-, and energy cycles. Dry organic matter (Org) has a lower heat conductivity (HCond) than mineral soil. Thus, high soil organic matter concentrations inhibit the heat transport into the ground, decreasing soil temperatures (Temp soil) below the layers near the surface. At the same time, they raise the maximum temperatures close to the surface which increases the heat loss due to the turbulent exchange with the atmosphere and the outgoing long-wave radiation—both of which have a non-linear dependency on the surface temperature. The higher porosity of organic matter also increases the soil’s water holding capacity and infiltration rates, which raises the water availability within the root zone (H_2_O). This increases transpiration and evaporation rates (Evapotrans) and lowers water stress-related limitations on productivity (NPP). A higher productivity in turn entails larger carbon inputs and higher concentrations of organic matter in the soil. The increase in evapotranspiration decreases the Bowen ratio and leads to a substantial cooling at the surface which further reduces the heat flux into the ground. The lower soil temperatures can have diverging effects on the moisture content of the soil (H_2_O). Permafrost (Perm) acts as a barrier that inhibits drainage, resulting in a higher overall water contents. At the same time, lower temperatures lead to a smaller fraction of the soil water being liquid and available to plants and microbes. The result is that close to the surface the plant available water predominantly increases while deeper within the soil a larger fraction of the water is frozen over longer periods, inhibiting microbial decomposition (Resp). The smaller decomposition rates lower the availability of nitrogen (Nitro) as they reduce the nitrogen mineralization in the soil. This in turn limits productivity and, consequently, the carbon input into the soil which further reduces soil decomposition. On average, the effects of a reduced nitrogen availability outweigh the effects of the higher water availability. As a result the soil stabilizes not only at a higher soil organic matter concentration, but also at (predominantly) lower in- and outgoing carbon fluxes.

**Fig. 6:**
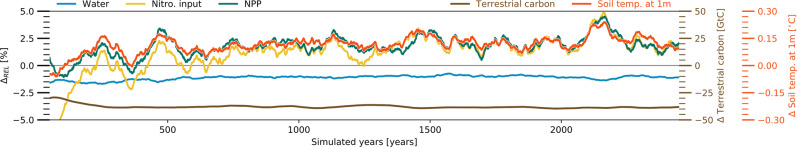
Long-term effects of an temperature overshoot on the high-latitude water-, energy-, and carbon cycle. Differences between simulations initialized with the soil carbon concentrations after (P2100) and before (NoOS) an temperature overshoot (OS) that persist under nontransient atmospheric conditions (PACT1.5). Shown are relative differences in total soil water content (blue line; left *y*-axis), inputs of mineral nitrogen (yellow line; left *y*-axis) and net primary productivity (green line; left *y*-axis) as well as absolute differences in total terrestrial carbon (soil organic matter and vegetation biomass; brown line; right *y*-axis) and MJJASO temperatures at a depth of 1 m (red line; right *y*-axis). With respect to soil water, nitrogen inputs, productivity, and temperature the figure shows the average over the permafrost-affected regions, while the terrestrial carbon is an accumulated value. The simulations were initialized with different carbon pools and different states of the vegetation, but they started from the same initial conditions with respect to the system’s physical state (NoOS). Hence, the differences indicate only the indirect effects of the OS. All lines show 100-year-running means. It should be noted that in individual grid cells, the simulations may not have reached a steady state within the investigated time-span of 2500 years (see Fig. 4), but when averaged over the entire permafrost region the differences between the simulations stabilize after roughly 1000 years, providing an order-of-magnitude estimate of the consequences of a temporary warming that are irreversible under nontransient conditions.

Organic matter acts as an insulation that impedes the ground heat flux during summer. A reduced vertical heat transport raises the annual maximum temperatures at the surface, but reduces them in the subjacent layers. Averaged over the entire year, the effect of SOM is a cooling of the entire soil column. Additionally, the hydrological properties of organic matter increase the available rootzone soil moisture, which raises transpiration and evaporation rates (Fig. 5). The latter leads to an evaporative cooling at the surface that further reduces the heat transport into the ground. Consequently, soil temperatures are notably higher when the nontransient atmospheric conditions are reached subsequent to an OS that reduces the soil carbon concentration (Fig. 6). The higher temperatures result in a smaller near-surface permafrost volume which affects the sub-surface hydrology and the soil water content. The soil temperatures and the water availability, in turn, control the vegetation’s primary productivity and the microbial decomposition rates, with the latter largely determining the SOM concentration at which carbon inputs and soil respiration reach an equilibrium. In our simulations, the OS-induced loss of organic matter entails hydrological soil properties that predominantly decreases the vegetation’s productivity, while the warmer soils increase decomposition rates. This stabilizes the in- and outgoing carbon fluxes at a lower SOM concentration, partly conserving the causative differences in the soil properties.

At the same time the higher soil respiration rates increase the amount of plant available nitrogen, as a large part of the mineral nitrogen stems from the mineralization of organic nitrogen during decomposition at low C:N ratios. Consequently, the vegetation is notably less affected by nutrient limitations when the PACT1.5 is reached subsequent to the OS, resulting in a higher productivity and larger carbon inputs into the soil. The latter allow to maintain the higher respiration rates and, consequently, higher levels of mineral nitrogen in the soil. In our simulations we find that, on average, the higher nitrogen availability outweighs the effects of limited water availability, resulting in larger in- and outgoing soil carbon fluxes. Due to these feedbacks, the consequences of a temporary warming in the high latitudes are not only long lasting, but in fact irreversible under nontransient atmospheric conditions, leading to higher below ground temperatures, increased soil respiration and a higher productivity at lower SOM concentrations.

**Fig. 7:**
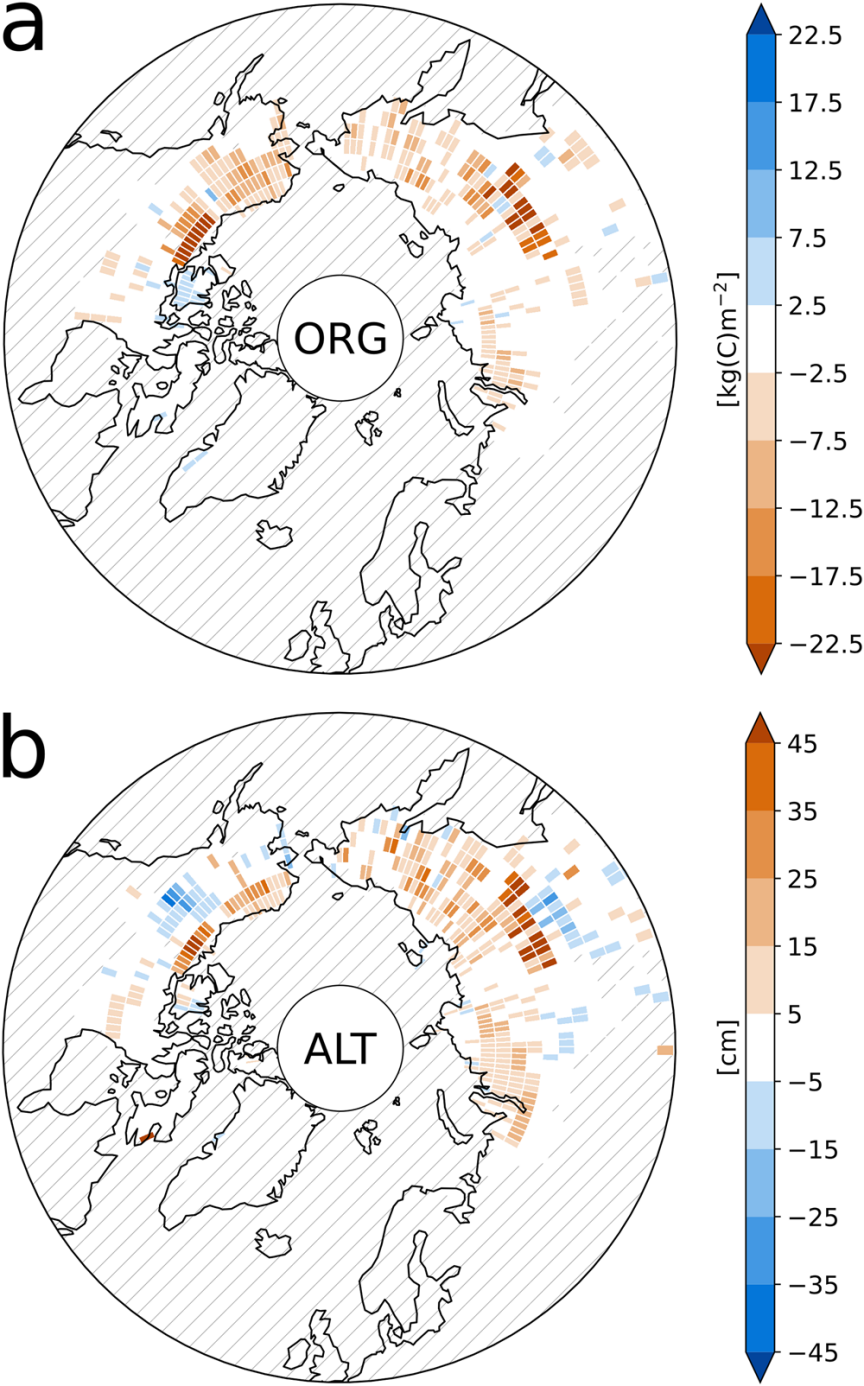
Regional differences in the legacy effects of an temperature overshoot. **a** Differences in carbon densities (ORG) between the simulation initialized with the soil carbon concentrations subsequent to an temperature overshoot (P2100) and the simulation starting from the carbon pools before the overshoot (NoOS) after 2500 years under PACT1.5 conditions (averaged over the simulated years 2000–2500). **b** Same as **a**, but for the differences in active layer thickness (ALT). Hatches mark non-permafrost grid cells.

With average temperature differences of about 0.1–0.2 °C, relative differences in the total soil water content, net primary productivity, and soil respiration of about 1–2.5 % and differences in the total terrestrial carbon of 40 GtC, the legacy effects of an OS may not appear dramatic. Here, it should be pointed out that, in our simulations, the above feedbacks do not cover the entire permafrost region, but that they appear to only be relevant for parts of the northern high latitudes, predominantly those that featured high SOM concentrations upon climate stabilization. Thus, while the southern permafrost regions in Eurasia show very little differences in the soil carbon concentrations after 2500 years under PACT1.5 conditions, the more northern regions and those on the American continent exhibit differences in the carbon densities in excess of 20 kg(C) m^−2^ (Fig. 7a). Furthermore, the above description constitutes a simplification that takes into account only the dominant processes and in some regions the feedbacks may be very different. For example, when largely water saturated, the heat capacity and conductivity of organic matter can exceed those of drier mineral soils which reduces the insulation at the surface. Consequently, the feedbacks have a high spatial variability and while we find the dominant effect of an OS to be warmer soils and a deeper active layer, there are areas where the near-surface permafrost volume is actually larger following the OS (Fig. 7b).

**Fig. 8:**
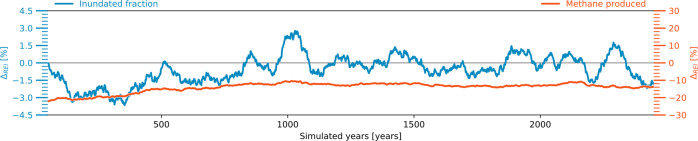
Long-term effects of an temperature overshoot on wetland formation and soil methane production. Differences between simulations initialized with the soil carbon concentrations after (P2100) and before (NoOS) an temperature overshoot that persist under nontransient atmospheric conditions (PACT1.5). Shown are relative differences in annual mean inundated area (blue line, left *y*-axis) as well as relative differences in the soil methane production (red line, right *y*-axes).

Finally, as the effects of an OS are more pronounced in regions with high carbon densities, they are likely to have stronger impacts on processes that are more prominent in organic-rich soils, such as the soil methane production. Here, our simulations indicate that the OS has a comparatively small effect on the average wetland area, however, it reduces the overall soil methane production by around 10%, because it changes the correlation between inundated soils and large SOM concentrations (Fig. 8). Thus, while the legacy effects of an OS may be confined regionally, they could have a pronounced effect on the high-latitude CH_4_-budget for millennia to come.

## Discussion

Large changes in the northern permafrost regions are already observable today—over the last decades, soil temperatures have increased by up to 2 °C, in many regions the active layer has deepened significantly and there has been a substantial retreat in the permafrost extent of the Arctic^6–15,22^. The modeling results presented here indicate that these observable changes do not depict the entire impacts due to the committed climate change. Even if all anthropogenic carbon emissions were stopped immediately and the climate stabilized instantly, it would take several centuries until the full impact of man-made warming on the high-latitude ecosystem becomes observable. But rather than reaching net zero emissions in the near future, it is probable that global mean temperatures will overshoot the PACT1.5 before they can be stabilized at this level, which—as our results have shown—will likely entail irreversible consequences for the state of soils and the terrestrial carbon cycle in the high northern latitudes. Here, our simulations do not indicate a threshold behavior—in which only extreme OS scenarios entail legacy effects, but rather that the long-term impact of an OS, on the permafrost region as a whole, is proportional to the OS’s magnitude (Supplementary Fig. 7). Consequently, small OSs could already alter the steady-state in the high northern latitudes, making the GHG emissions of even the next 15–30 years highly relevant for the long-term future of the Arctic.

It should be noted that the present experimental setup may have even lead to an underestimation of the OSs’ legacy effects. On the one hand we used the land surface model in an uncoupled setup, running it with prescribed atmospheric conditions, while, in reality, there is a number of local land-atmosphere feedbacks that have the potential to enhance these effects. A lower Bowen ratio, for example, means that more moisture and less sensible heat is transported to the atmosphere. This cools the boundary layer and raises the relative humidity, which in turn increases precipitation rates and the water availability, further reducing the Bowen ratio^47,48^. On the other hand, it is important to keep in mind the limitations of current generation Earth system models. While these models capture the fundamental physical and biophysical processes, they do not account for small scale hydrological and geomorphological mechanisms, both of which play a key role in the permafrost-affected landscapes. Here, Arctic ecosystems are especially challenging for two reasons. First, the interactions between biogeochemical and biogeophysical factors can vary at very fine spatial scales^49^. Second, Arctic landscapes are highly susceptible to non-linear and abrupt change processes^50–52^. These change processes are often triggered by events that are spatially and temporally very confined, such as local weather extremes, but they hold the potential to alter the face of entire regions. Prominent examples are geomorphological processes linked to the degradation of permafrost, including thermokarst features such as the formation of connected troughs through the preferential degradation of ice wedges in ice-rich permafrost^53–55^, thaw lake formation, and drainage^56^, as well as ground subsidence^49,50^. As these processes are not captured by large-scale models such as JSBACH, it remains an open question if the small scale geomorphological effects of an OS would be fully reversible. Here, it is plausible that ground subsidence, the melting of ice lenses or thermokarst features that occurred during an OS could entail or even constitute additional consequences that are irreversible under steady climate conditions.

Finally, our results are subject to large uncertainties, not only because of the coarse resolution of our model and the idealized setup of the study. More importantly, our results rely on simulations with just one land surface model, while studies have shown that the simulated dynamics in permafrost regions can vary substantially between models. There is even some disagreement on fundamental questions, such as whether the high latitudes will become wetter or dryer in a warmer climate^57^. Furthermore, due to the extensive timescales involved in our study, we had to rely on a single, best-estimate, parameter set for our simulations. This prohibits an uncertainty estimate with respect to our setup. However, from previous simulations with our adapted model we know that—while the general behavior of the model is robust—many important processes, such the soil methane emissions, are very sensitive to the parameter choices in the model^42^. Thus, our investigation can only provide order-of-magnitude estimates of the adjustment timescale or the magnitude of the legacy effects of an OS but not exact values. Nonetheless, our findings should serve as a warning that the emission pathway to climate stabilization could affect the state of the northern permafrost regions for thousands of years.

## Methods

All simulations for this study were performed using JSBACH, the land surface component of the Max-Planck-Institute for Meteorology’s Earth system model MPI-ESM1.2^41^. The standard version of this model includes certain parametrizations that are not well suited for the specific conditions that are characteristic of the northern high latitudes—most importantly it does not account for freezing and melting of water within the soil—which made it necessary to adapt the model to the requirements of this study. The adaptations include changes of JSBACH’s carbon cycle and soil-physics routines as well as the implementation of a new module that estimates the fractional cover of inundated areas and wetlands. In the following we will give a brief summary of these changes, while a more detailed description can be found in de Vrese et al.^42^. Furthermore, the methods section provides a concise overview over the simulations (summarized in Table 2) and certain aspects of the analyses that were performed in the context of this study.

**Table 2:** Overview of the simulations that were performed for this study.

Simulation	Temp. cover	Spatial cover	Forcing	*C*_init_	*P*_init_	Sec.	Fig.
NoOS	1850–2035	45–90°N	Historical + SSP5-8.5	Observ.	pi-control	2^*1^	1
P2050	2035–2065	45–90°N	Overshoot, SSP5-8.5 peak in 2050	NoOS	NoOS	2^*1^	1
P2075	2035–2115	45–90°N	Overshoot, SSP5-8.5 peak in 2075	NoOS	NoOS	2^*1^	1
P2100	2035–2165	45–90°N	Overshoot, SSP5-8.5 peak in 2100	NoOS	NoOS	2^*1^	1
CC ${}_{{{{\rm{NoOS}}}}}^{{{{\rm{NoOS}}}}}$	1000 years	45–90°N	^*3^PACT1.5 (SSP5-8.5: 2030–2040)	NoOS	NoOS	2^*1^	2, 3
CC ${}_{{{{\rm{P2050}}}}}^{{{{\rm{P2050}}}}}$	1000 years	45–90°N	^*3^PACT1.5 (SSP5-8.5: 2030–2040)	P2050	P2050	2^*1^	2, 3
CC ${}_{{{{\rm{P2075}}}}}^{{{{\rm{P2075}}}}}$	1000 years	45–90°N	^*3^PACT1.5 (SSP5-8.5: 2030–2040)	P2075	P2075	2^*1^	2, 3
CC ${}_{{{{\rm{P2100}}}}}^{{{{\rm{P2100}}}}}$	1000 years	45–90°N	^*3^PACT1.5 (SSP5-8.5: 2030–2040)	P2100	P2100	2^*1^	2, 3
CC ${}_{{{{\rm{NoOS}}}}}^{{{{\rm{NoOS}}}}}$	2500 years	45–90°N	^*4^PACT1.5 (SSP5-8.5: 2030–2040)	NoOS	NoOS	2^*2^	4, 6–8
CC ${}_{{{{\rm{NoOS}}}}}^{{{{\rm{P2100}}}}}$	2500 years	45–90°N	^*4^PACT1.5 (SSP5-8.5: 2030–2040)	P2100	NoOS	2^*2^	4, 6–8
CC-REG${}_{{{{\rm{NoOS}}}}}^{{{{\rm{NoOS}}}}}$	7500 years	Grid box	^*4^PACT1.5 (SSP5-8.5: 2030–2040)	CC ${}_{{{{\rm{NoOS}}}}}^{{{{\rm{NoOS}}}}}$	CC ${}_{{{{\rm{NoOS}}}}}^{{{{\rm{NoOS}}}}}$	2^*2^	4
CC-REG${}_{{{{\rm{NoOS}}}}}^{{{{\rm{P2100}}}}}$	7500 years	Grid box	^*4^PACT1.5 (SSP5-8.5: 2030–2040)	CC${}_{{{{\rm{NoOS}}}}}^{{{{\rm{P2100}}}}}$	CC${}_{{{{\rm{NoOS}}}}}^{{{{\rm{P2100}}}}}$	2^*2^	4

Rows indicate the temporal- and the spatial coverage of the simulations as well as the atmospheric forcing that was used. Furthermore, they indicate the origin of the initial soil carbon pools (*C*_init_) and the physical state that was used to initialize the model (*P*_init_). Finally, the table gives the section in which the simulation is analyzed and the figures in which it is shown.

^*1^Subsec.: “Adjustment timescales in permafrost regions”.

^*2^Subsec.: “Multistability in high-latitudes and legacy effects of overshoot scenarios”.

^*3^Cyclic forcing.

^*4^Forcing year is randomly selected from forcing period.

### Model

#### Soil carbon dynamics

In JSBACH, the soil carbon dynamics are simulated by the YASSO model, which determines decomposition rates using quality-dependant mass loss parameters that are scaled to account for the temperature and moisture dependencies of the decomposition process^58,59^.

In the standard model, the scaling factors are based on the surface temperature and precipitation rates. However, this approach is problematic for permafrost-affected regions, where soils store large amounts of organic matter in depths of several meters. Here, the conditions under which organic matter decomposes are only poorly represented by surface temperatures and precipitation rates. In order to adapt the model’s soil carbon dynamics to these conditions, we maintained the original separation into different lability classes, but implemented a vertical discretization of the below ground carbon pools. This allows us to calculate the respective decomposition rates using the depth dependant soil temperature and liquid soil water content (see below). As the present model version distinguishes between anoxic and oxic decomposition, we additionally separate the organic matter which is stored within inundated soils from the soil carbon in the non-inundated part of a grid box. At the surface, we employ the same decomposition rates as the standard YASSO model, with the exception that we assume the soil carbon in the inundated grid box fraction to decompose at a different rate than the carbon in the non-inundated fraction. For the below ground decomposition rates, we included several (optional) moisture and temperature dependencies^60^. In the present study, we chose the temperature dependency parametrization of the YASSO model—using soil- instead of the surface temperatures—in combination with a simplified version of the moisture limitation function used in the CENTURY ecosystem model^61^.

To distribute the soil carbon on the vertical layers, the carbon inputs are matched to plant-(functional)-type-specific root-profiles^62^, assuming that the inputs on a given layer are proportional to the fraction of the roots that are located within this layer. These inputs consist of root exudates and the litter flux from the below ground vegetation biomass, with the latter including the flux due to disturbances such as fires or strong winds. Furthermore, we included a scheme to account for the accumulation of organic matter at the surface and its vertical transport due to bio- and cryoturbation. For the vertical transport we largely follow the approach of Koven et al.^63,64^ and described the vertical mixing of SOM as a diffusive transport. However, instead of assuming uniform mixing rates throughout the permafrost affect regions, the transport velocities are affected by two factors representing the grid box-specific saturation of the active layer and the number of days in which temperatures crossed the freezing point to include the effect of repeated freeze-thaw cycles.

#### Representation of physical processes

Our implementation of the physical processes that are especially relevant in the high latitudes is a modified version of the approach proposed Ekici et al.^43^ who introduced a five-layer snow scheme, the phase change of water within the soil and the effect of water on the soil’s thermal properties.

To account for the impact of organic material on the soil’s thermal and hydrological properties Ekici et al.^43^ introduced a pervasive organic-top-soil-layer, which captures the respective effects only at the surface and is independent of the simulated carbon pools. As our model represents the vertical structure of the SOM explicitly, we extended this approach and determine a given soil property at a given depth based on the properties of soil organic and mineral matter and the respective (volumetric) fractions—resulting in interactive, depth-dependent soil properties. For many soil properties, such as the porosity, this was done by a simple weighted average but other key variables, such as the heat conductivity, have a non-linear dependency on the organic fraction as they are derived from several other soil properties (for an overview of the key dependencies see Supplementary Fig. 4).

The approach by Ekici et al.^43^ accounts for the fraction of the soil water that remains liquid at sub-zero temperatures, the so called supercooled water. Our approach also allows a given fraction of the water to remain liquid, but different to Ekici et al.^43^ we assume that this water can not move through the soil as it only forms as a very thin film around the solid soil particles (note that the model does not account for chemical factors that lead to the formation of supercooled water). Furthermore, we inhibit the movement of water through frozen soil layers, if more than half of the respective pore space is occupied by ice. Finally, the standard model allows lateral drainage (subsurface runoff feeding the river discharge) from all soil layers located above the bedrock. These fluxes are included to account for the effects of connected vertical channels in coarse material or cracks and crevices which are assumed to be present in all grid cells at the coarse resolution of the model. In addition to percolation, these connected pathways transport the water deeper underground toward the bedrock boundary where it runs of as base flow. In the present model version, we assume that, in permafrost regions, these vertical channels would predominantly be blocked by ice, allowing lateral drainage only from the soil layer in which the bedrock starts or from those below which the soil is fully saturated.

At the surface, we changed the conditions controlling infiltration from a (partly) temperature-dependant- to a saturation-dependent formulation, allowing a fraction of the snow melt to infiltrate into the soil. Finally, we changed the water stress formulations which controls transpiration rates. JSBACH employs a fixed root-depth to calculate the relative saturation of the root zone and the plant’s water stress. As a consequence, plants in some regions extend their roots into the permafrost, resulting in a constant water stress that reduces transpire rates even when the near-surface soil layers are largely water saturated. In reality however the vegetation could adapt by developing shallower roots. For the present model version we changed this approach and calculate the water stress relative to the part of the root zone that is located above the permafrost table.

#### Wetlands and inundated areas

The standard version of the model does not account for surface water bodies or inundated areas and, for the present study, we implemented two schemes that represent different aspects of their formation. The first scheme simulates the effect of ponding—the formation of wetlands because water neither infiltrates nor runs off but pools at the surface—while the second scheme uses a TOPMODEL-based approach to account for inundated areas that form in highly saturated soils, due to low drainage fluxes^65^. In the model, wetlands affect infiltration, drainage, and evaporation rates, the vegetation cover and the surface albedo while the extent of saturated soils is only used for a diagnostic subgrid-scale distribution of the soil moisture, required to distinguish between areas where organic matter decomposes under oxic and anoxic conditions. Note that our setup does not contain peatland-specific parametrizations, such as vegetation types that support the formation of bogs. However, especially in western Siberia the model simulates a large wetland area which helps maintain the characteristic soil carbon concentrations, because a large fraction of the organic matter decomposes only slowly under anoxic conditions. Furthermore, the model simulates strong nitrogen limitations predominantly in those regions that have a high fraction of inundated areas, which agrees with high-latitude peatlands often being nutrient limited.

### Setup and analysis

#### General setup

The aim of the present study is to investigate the behavior of the land surface under very specific atmospheric conditions. To this end, we use JSBACH in an offline-setup, in which the model is not coupled to the atmosphere but driven by either observations or output from the fully-coupled ESM (wind speed, atmospheric temperature, and humidity at a height of roughly 30 m as well as radiative fluxes and precipitation rates) and prescribed atmospheric GHG concentrations.

In all simulations, JSBACH is forced with output from simulations with the standard version of the MPI-ESM1.2 that were performed in the context of the 6th phase of the Coupled Model Intercomparison Project (CMIP6)^66^. These simulations cover the historical period—1850 to 2014—and a scenario period ranging between the years 2015 and 2100. All future climate trajectories that are investigated in this study are based on the Shared Socioeconomic Pathway 5 and the Representative Concentration Pathway RCP8.5 (SSP5-8.5)^67,68^. SSP5-8.5 targets a radiative forcing of 8.5 Wm^−2^ in the year 2100 and assumes the atmospheric CO_2_ concentrations to increase to about 1000 ppmv by the end of this century. In this scenario, the global mean temperature increases to about 4 °C above preindustrial levels and the precipitation rates in the high northern latitudes to about 675 mm year^−1^. There are no scenario simulations available that could provide the forcing for a decrease in GHG concentrations and, for the OS scenarios, we assume that the decrease simply reverses the trajectory of the increase prior to the peak. In simulations with the MPI-ESM1.2, SSP5-8.5 reaches the PACT1.5 around the year 2035. For simulations that target stable PACT1.5 conditions, we use the atmospheric forcing from the period 2030 to 2040.

All simulations have the same general setup, with a horizontal resolution of T63 (1.9° × 1.9°), which corresponds to a grid-spacing of about 200 km in tropical latitudes, a temporal resolution of 1800 s and a vertical resolution of 18 sub-surface layers that reach to a depth of 100 m, 11 of which are used to represent the top 3 m of the soil column (note that this vertical resolution is not the standard configuration of the model. In contrast, for the model comparison in Supplementary Figs. 1–3, the standard model and the version of Ekici et al.^43^ were run using the standard vertical five-layer grid). We ran most of the simulations covering the region north of 45°N, with the exception of the CC-REG${}_{{{{\rm{NoOS}}}}}^{{{{\rm{NoOS}}}}}$ and CC-REG${}_{{{{\rm{P2100}}}}}^{{{{\rm{NoOS}}}}}$ simulations (see below), where we simulated only selected grid boxes.

To be able to start our simulations with reasonable carbon pools we followed an approach that aims to minimize the inconsistency between observed soil carbon concentrations, the soil depths at the model’s coarse resolution and the simulated climatic conditions. As observational basis for the initial soil carbon pools, we use data from the WISE30sec dataset^69^—above a depth of 2 m—and data from the Northern Circumpolar Soil Carbon Database (NCSCDv2)^21^—for depth between 2 and 3 m. Below this depth the soil is initialized without organic matter, neglecting possibly up to 650 GtC contained in Yedoma or deltaic alluvium^21^.

When limiting the organic matter to the top- and subsoil—the soil above the bedrock—of the standard setup, the model is initialized with as little as 636 GtC of organic matter in the northern high latitudes instead of the 1015 GtC that the combination of WISE30sec- and NCSCDv2 data provides for the uppermost 3 m. For the present setup, we combined two approaches to mitigate the problem of underestimated soil carbon pools. On the one hand we extended the soil depths in the model based on soil parameters that had previously been used by Schneider von Deimling et al.^34,70^, which allows to store more organic matter—about 797 GtC—in the appropriate regions. Furthermore, we up-scaled the carbon pools that are located above the bedrock boundary to obtain an overall carbon content that is closer to observations—about 931 GtC. The datasets contain no information with respect to the quality of the SOM and we used the equilibrium distribution (amongst quality classes) that is simulated by the MPI-ESM1.2 at the end of the standard pi-control simulation performed in the frame of CMIP6.

We initialize the first simulation—leading up to the year 2035—with the observation-based, present-day carbon pools but start it at the end of the preindustrial period. In the high northern latitudes this allows the carbon concentrations within the (simulated) active layer to adapt to the simulated climatic conditions and vegetation dynamics, while the perennially frozen regions of the soil conserve the observed carbon concentrations. By initializing the simulation in this manner, the carbon pools at the beginning of the twenty-first century are largely in agreement with observations, while there are no implausible soil carbon emissions that result from the mismatch between real-world and simulated active layer depths. In all other simulations, the carbon pools are initialized using simulated soil carbon concentrations (see below).

#### Simulations

The study targets the behavior of the northern permafrost-affected regions under stable atmospheric conditions. In an initial step this required determining the system’s physical state (*P*_init_) as well as the vegetation cover and the soil carbon concentrations (*C*_init_) at the beginning of the stable period. Here, we started the first simulation (NoOS) in the year 1850 at the end of the preindustrial (pi) period, with observation-based *C*_init_ (see description of initial carbon pools) and *P*_init_ taken from a standard pi-control simulation (see Table 2). This simulation was run until the year 2035 using standard output from CMIP6 experiments—historical and SSP5 scenario simulations—to force the model (see Supplementary materials Fig. 5). Subsequently, we made three simulations for the OS scenarios which started in the year 2035 with *C*_init_ and *P*_init_ taken from the last year of the NoOS simulation. We simulated a small OS with a peak in the year 2050, in which the forcing is reversed to PACT1.5 levels by 2065 (P2050), a moderate OS peaking in 2075, with a reversal of the forcing by 2115 (P2075) and an extreme OS peaking in 2100 and ending in 2165 (P2100).

The final states of the four OS simulations (including NoOS) were then used to initialize four simulations in which the atmospheric conditions were maintained at the PACT1.5 level for 1000 years (CC${}_{{{{\rm{NoOS}}}}}^{{{{\rm{NoOS}}}}}$, CC${}_{{{{\rm{P2050}}}}}^{{{{\rm{P2050}}}}}$, CC${}_{{{{\rm{P2075}}}}}^{{{{\rm{P2075}}}}}$ and CC${}_{{{{\rm{P2100}}}}}^{{{{\rm{P2100}}}}}$). As these simulation were initialized with both, *C*_init_ and *P*_init_, taken from the respective OS scenario they include the OS’s indirect as well as the OS’s direct effects. Note that these simulations were forced by cycling the forcing of the 2030–2040 period.

To determine the OS’s indirect effects—resulting from changes in the soil’s hydrological and thermal properties—we made an additional simulation (CC${}_{{{{\rm{NoOS}}}}}^{{{{\rm{P2100}}}}}$) that was initialized with *C*_init_ taken from the extreme OS scenario (P2100) but *P*_init_ taken from the no-OS scenario (NoOS). This simulation was run for 2500 years under PACT1.5 conditions and, for comparison, we also extended the CC${}_{{{{\rm{NoOS}}}}}^{{{{\rm{NoOS}}}}}$ simulation to 2500 years. Note that these simulations were forced by randomly selecting a forcing year from the 2030 to 2040 period.

Finally, we found that even after 2500 years under PACT1.5 conditions, many grid boxes had not reached an equilibrium state. Hence, to determine whether the initial soil carbon concentration truly affects the steady-state we extended both CC${}_{{{{\rm{NoOS}}}}}^{{{{\rm{NoOS}}}}}$ and CC${}_{{{{\rm{NoOS}}}}}^{{{{\rm{P2100}}}}}$ by another 7500 years (CC-REG${}_{{{{\rm{NoOS}}}}}^{{{{\rm{NoOS}}}}}$ and CC-REG${}_{{{{\rm{NoOS}}}}}^{{{{\rm{P2100}}}}}$). However, the simulations were only extended in a few selected grid boxes.

#### Analysis

The analyses in this study are straightforward but there are certain aspects that need to be clarified either because they involve non-standard measures—i.e., the adjustment timescale—or because they have a distinct impact on the results—i.e., the length of simulations and the spatial aggregation of the results.

It can take several thousand years until certain below ground variables stabilize under a given nontransient atmospheric forcing. However, the respective trends are often extremely small. Thus, for our analyses we defined the adjustment timescale as the duration until a given variable has largely adapted to the atmospheric conditions and the respective trend has fallen under a certain threshold. More precisely we used the point in time at which the rate of change of a given variable (based on 100-year running means) has decreased by an order of magnitude relative to the rate of change during the first 100 years of the simulation. To make the timescales comparable between simulations, we applied the same threshold for all the simulations, using the rate of change during the first 100 years of the NoOS simulation.

In the present study we analyzed model output over periods of up to 10,000 years and it should be noted that JSBACH has certain shortcomings with respect to periods ⪆1000 years. Most importantly, the model does not account for pedogenesis—e.g., the properties of the mineral soil fraction are constant, the model does not include soil erosion or dust depositions and the soil depths are fixed—which becomes increasingly important on these long timescales. Thus, while the model represents the build up or loss of organic matter in the soil, the simulations may miss certain aspects of the long-term sub-surface dynamics that play a role for the soil composition. Nonetheless, the model is capable of showing that these dynamics can be fundamentally different for different initial conditions—here the initial SOM concentrations.

The northern high latitudes feature very diverse landscapes and the local response to increasing and decreasing temperatures can differ substantially, depending on a number of factors. Nonetheless, detailed regional analyses are beyond the scope of our study and we mainly focus on the northern permafrost region in its entirety. It should be noted that the spatial aggregation has important effects for the results of this study: for the permafrost-affected region as a whole we find changes to occur gradually and without obvious temperature thresholds. However, when looking at individual grid boxes we often find changes to be abrupt and especially permafrost degradation can occur very fast, with soils desiccating in a matter of a few years and decadal temperature changes in the order of degrees.

## Supplementary information

Supplementary Information

## Data Availability

The primary data are available via the German Climate Computing Center’s long-term archive for documentation data (https://cera-www.dkrz.de/WDCC/ui/cerasearch/entry?acronym=DKRZ_LTA_903_ds00001).

## Code availability

The model and scripts used in the analysis and other Supplementary information that may be useful in reproducing the authors’ work are archived by the Max Planck Institute for Meteorology and can be obtained by contacting publications@mpimet.mpg.de. The code is subject to the license terms of the MPI-ESM license version 2 and will be made available to individuals and institutions for the purpose of research.
