# Supplementary material for: Timescales of the permafrost carbon cycle and legacy effects of temperature overshoot scenarios
Source: Nat Commun. 2021 May 11;12:2688. doi: 10.1038/s41467-021-23010-5 (PMC8113593; doi:10.1038/s41467-021-23010-5)
Supplement: Supplementary file 1 — Supplementary Information [file 41467_2021_23010_MOESM1_ESM.pdf]

Supporting Material: Timescales of the permafrost carbon cycle and legacy effects of temperature overshoot scenarios

Philipp de Vrese[1,*] **and Victor Brovkin**[1,2]

[1]Max Planck Institute for Meteorology, The Land in the Earth System, Hamburg, 20146, Germany
[2]University of Hamburg, Center for Earth System Research and Sustainability, Hamburg, 20146, Germany
[*]philipp.de-vrese@mpimet-mpg.de

Supplementary Figures

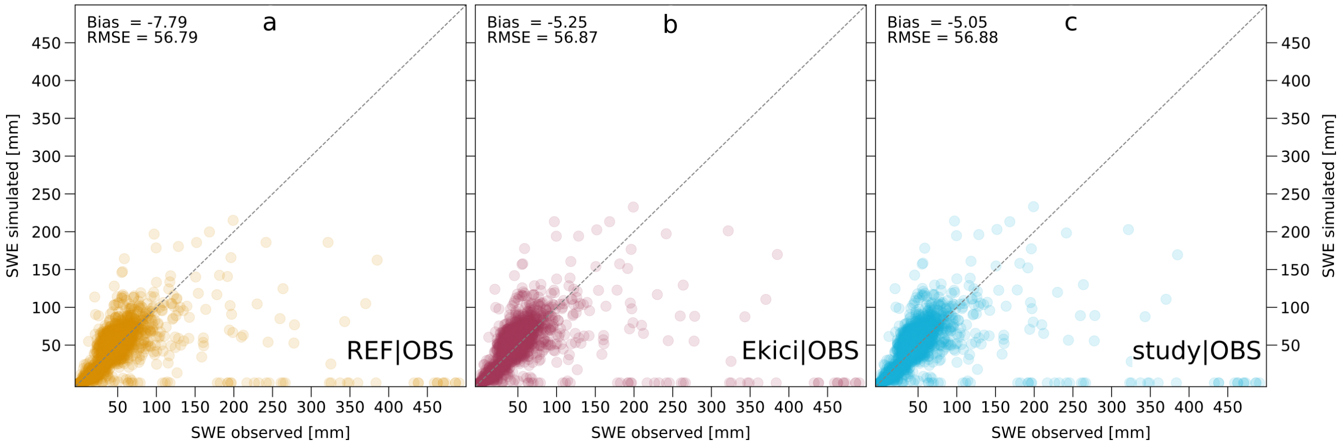

Supplementary Figure 1. Comparison of simulated and observed snow water equivalent in the northern high latitudes
Panels a - c show the simulated annual mean snow water equivalent (SWE) in grid cells north of 47.5°N and corresponding data from the Northern Hemisphere subset of the Canadian Meteorological Centre operational global daily snow depth analysis[1]. For the comparison, the observational data was remapped to the model-resolution of T63 and averaged over the observed 1998 - 2012 period. a) Observed and simulated SWE, for the standard JSBACH model. Note that, with observed annual mean SWEs of up to 2500 mm, there are a few grid boxes (23 out of 1723) in which the observed SWEs far exceed the simulated range. These extreme values are not included in the analysis and accounting for the respective data increases the model bias and root mean square error (RMSE) substantially (bias: -18.01; RMSE: 116.97). b) Same as panel a but for the model version of Ekici et al. (2014)[2,3] (including all data: bias: -15.44; RMSE: 116.67). c) Same as panel a but for the setup used in the present study (including all data: bias: -15.23; RMSE: 116.65).

The simulated snow cover is largely determined by the precipitation rates and the simulated surface temperatures. Especially the later are captured well by the latest release of the MPI-ESM[4], thus also the simulated SWEs compare reasonably well with observations when the model is run with the forcing data derived from MPI-ESM model output. It is clear that the standard model (a) over- and underestimates the SWE for individual grid boxes – resulting in a RMSE of 57 mm – and that there is even a number of cells in which the model simulates no lasting snow cover, while the observations indicate a large SWE. However, with a SWE bias of less than -8 mm (-18 mm when including all data points), the model captures the conditions at the surface sufficiently well, especially given the highly idealized scenarios investigated in the present study. The model adaptations done by Ekici et al. (2014), including the introduction of the new 5-layer-snow scheme, change the simulated SWE only slightly (b): The mismatch in individual cells remains high – indicated by an RMSE of 57 mm –, while the overall bias is reduced to -5 mm. The same is true for the model version used in the present study, and bias and RMSE are almost the same as in simulations using the model setup by Ekici et al. (2014).

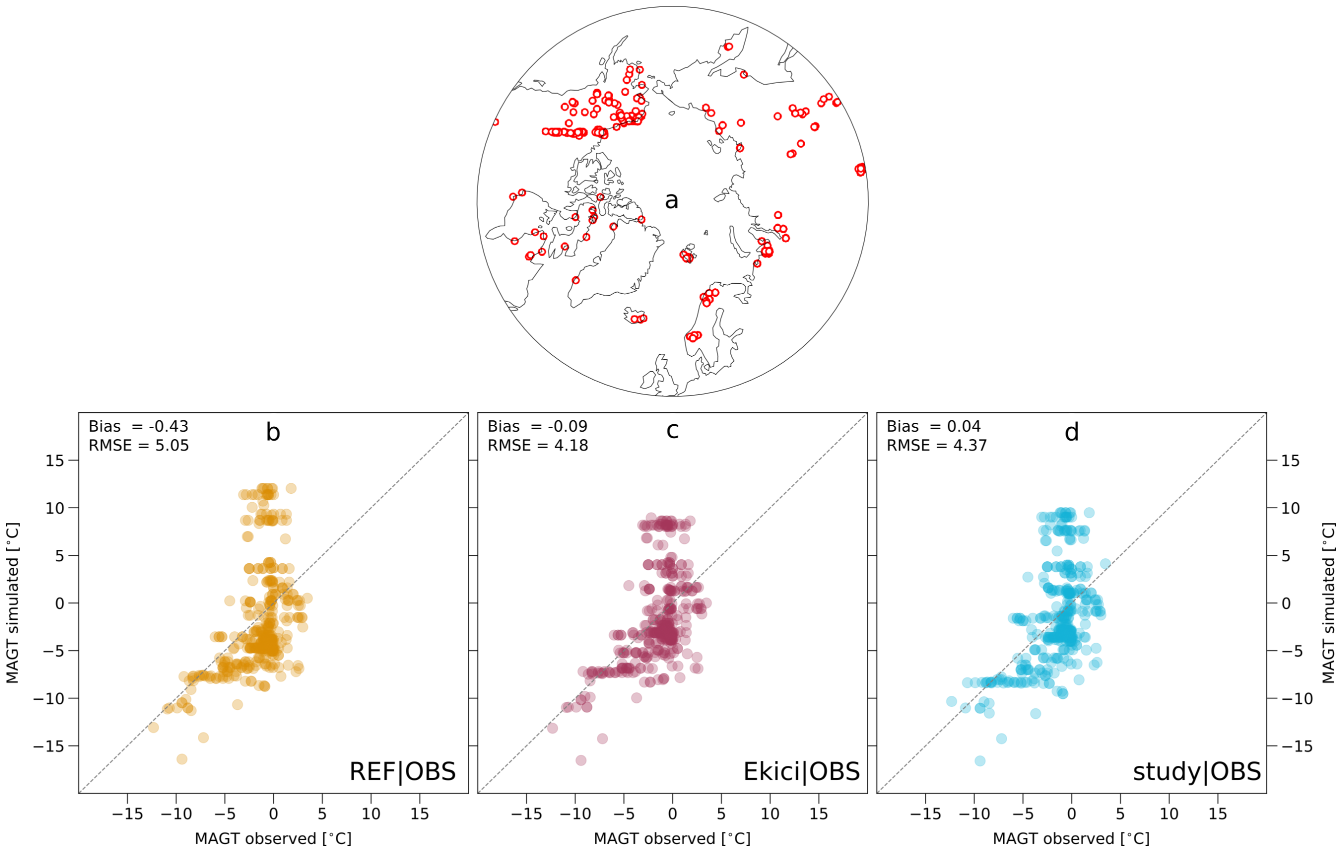

Supplementary Figure 2. Comparison of simulated and observed mean annual ground temperatures
a) Locations of borehole measurements provided by the Global Terrestrial Network for Permafrost initiative[5–7]. The sites provide measurements for different years during the period 1970 - 2010 – from which we consider only those from the period 2000 - 2010 – and give the mean annual ground temperature (MAGT) at different depths, ranging between 0.3 m and 100 m – here we only consider data from depth between 5 m and 20 m. b) Comparison of observed mean annual ground temperature (MAGT) and simulated annual mean soil temperatures at the lowest model level (in the standard vertical resolution reaching to a depth of 10 m) for the standard JSBACH model. The simulated MAGT constitute the mean of the period 2000 - 2010. c) Same as panel b but for the version by Ekici et al. (2014)[2,3]. d) Same as panel b but for the model used in the present study (here, temperatures correspond to a depth of 11 mn).

Using the fully-coupled MPI-ESM, the simulated MAGTs in the northern high latitudes are predominantly closer to the lower end of the observational range[8]. This is also the case for JSBACH-standalone simulations, when the standard model is driven by MPI-ESM output (b). For lower observed MAGTs (MAGT $<$ -5°C), the standard model systematically underestimates the temperatures, while MAGTs closer to the freezing point (-5°C $<$ MAGT $<$ 5°C) are similarly over- and underestimated, resulting in an RMSE of 5.05°C and a bias of -0.43°C. In simulations with the model version of Ekici et al. (2014), the average MAGT is slightly higher and the partial over- and underestimation of MAGTs closer to the freezing point is reduced. Overall, this brings the simulated MAGT closer to the observed values – indicated by a reduction of the RMSE to 4.18°C – and reduces the temperature bias to -0.09°C. In simulations with the model version used in the present study, the lower MAGTs match the observations slightly better, or rather they are less systematically underestimated, which reduces the temperature bias even further to 0.04°C. However, for the MAGTs closer to the freezing-point there is no notable improvement and the RMSE slightly higher than that of the model version by Ekici et al. (2014). There are strong indications that the large differences at individual sites are partly resolution dependent, and when the model is run with observation-based forcing at a 0.5° resolution, the RMSE is substantially smaller, while the bias is comparable[2,9,10]. Nonetheless, there are also larger areas in Eastern Siberia (Yakutia) where simulations with JSBACH may exhibit a cold bias of several degrees, but where only few observational sites exist. This potential bias appears to exist in all model versions independent of the forcing or the spatial resolution[2,9,11]. Such a cold bias would not be unique to JSBACH and could be related to the zero-flux assumption at the bottom boundary of the soil column – which is shared by most land surface models – or to an underestimation of the snow cover[2,9]. However, when accounting for the general warm bias and the uncertainty in the available reference data for Yakutia[12], JSBACH's cold bias becomes almost negligible, being prominent only in mountainous regions[9]. Here, the representation of the snow cover is particularly challenging as the latter depends on the local orography which is difficult to represent in coarse-resolution models. Finally, even though the MAGT is very similar for the three model versions, the magnitude of the annual temperature cycle is substantially different, which has important consequences for the simulated active layer depths (see Fig. 3 below).

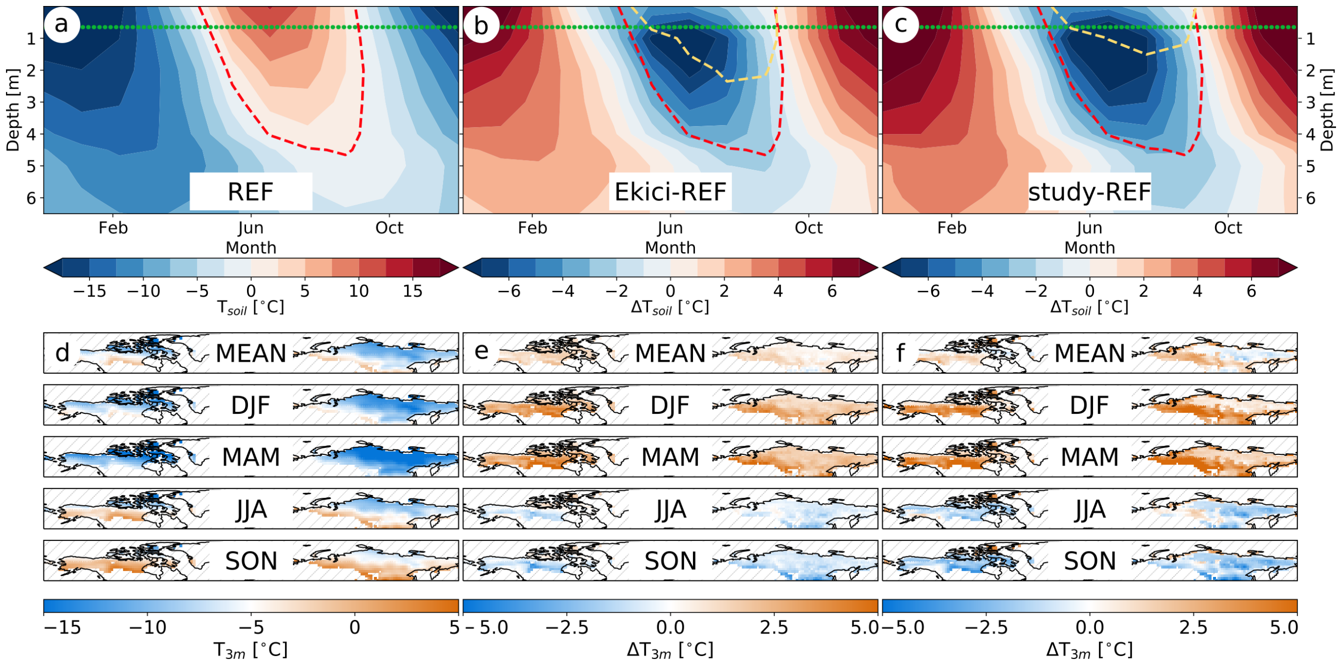

Supplementary Figure 3. Comparison of simulated soil temperatures for different JSBACH setups
a) Simulated soil temperatures, using the standard JSBACH model, averaged across the northern permafrost regions. Shown is the annual cycle averaged over the period 1990 - 2010. The (dashed) red line highlights the position of the zero-degree-threshold and the (dotted) green line shows the observed end-of-the-season thaw depths averaged across the sites of the Circumpolar Active Layer Monitoring program[13] that are located in the simulated permafrost domain. It should be noted that the maximum depth of the zero-degree-threshold and the average end-of-the-season thaw depth can only be used for a rough comparison, as the latter is based on data from stations that are not evenly distributed across the permafrost domain, introducing a slight bias towards a higher permafrost table. b) Difference between simulated soil temperatures using the model setup by Ekici et al. (2014)[2,3] and the standard model. The (dashed) red line shows the zero-degree-threshold for the standard model and the yellow line that of the Ekici-version. c) Same as panel b but for the model version used in the present study and the standard model. d) Simulated soil temperatures at a depth of 3 m, using the standard JSBACH model. Shown are (top to bottom) the annual mean, December - February mean, March - May mean, June - August mean and the September - November mean. e) Difference between simulated soil temperatures at a depth of 3 m using setup by Ekici et al. (2014) and the standard model. f) Same a panel e but for the model version used in the present study and the standard model.

When simulated with the standard model (a,d), the soil temperatures in the permafrost region exhibit a pronounced seasonal cycle throughout the entire soil column. At the surface, the spatially averaged temperatures range between roughly -15°C and 10°C, and at a depth of more than 4 m the annual temperature cycle still has an amplitude of about 15°C. Here, temperatures during (boreal) summer and fall are comparatively high and positive values reach (on average) beyond a depth of 4 m. As a result the standard model overestimates the thickness of the active layer substantially, despite the simulated mean annual ground temperatures being closer to the lower end of the observed range[8]. In the model version of Ekici et al. (2014) the amplitude of the annual temperature cycle is reduced and especially the 5-layer-snow scheme, accounting for the phase transition of water in the soil and incorporating the insulating effect of an organic topsoil layer dampen the temperature oscillations substantially (b,e). During winter, the near-surface layers are on average up to 7°C warmer, while the high temperatures during summer and fall are reduced correspondingly. The latter are particularly important for the active layer thickness and, on average, positive temperatures are limited to the uppermost 2 m of the soil column. For this model version the representation of the active layer is strongly improved, but there is still a tendency to overestimate the maximum thaw depths[2,3,14]. In simulations with the model version used in the present study, the insulating effect of organic matter is more pronounced – as organic matter is not only considered in the uppermost soil layer but also in lower layers – which further reduces the amplitude of the annual temperature cycle (c,f). Consequently, the soil temperatures during summer and fall are colder and, on average, positive values only extend to a depth of about 1 m. For this model version, the simulated annual maximum thaw depths are very close to the observed end-of-the-season thaw depths that have been reported for sites located in the simulated permafrost domain[15].

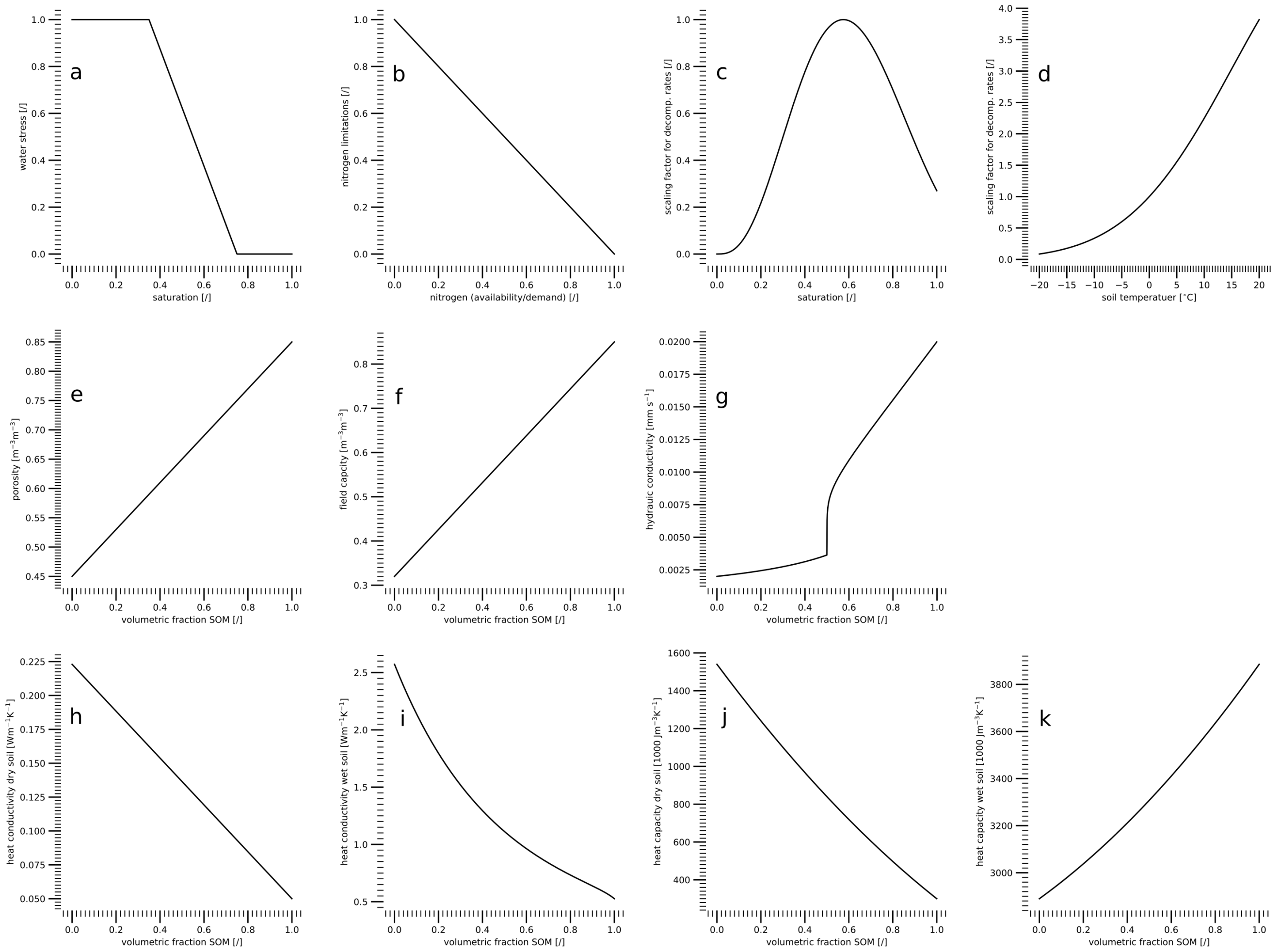

Supplementary Figure 4. Dependence of key processes and properties on the state of the soil and the soil organic matter content

a) NPP-limitation as a function of (liquid) soil water availability, b) relative reduction of productivity and respiration rates as a function of nitrogen availability, c) scaling factor for soil decomposition rates as a function of (liquid) water availability (the scaling factors are multiplied with the lability-class dependent reference decomposition rates) and d) scaling factor for soil decomposition rates accounting for the temperature dependency of the process. e-k) Examples of dependencies of soil properties on the SOM content (note that the actual values vary from grid cell to grid cell depending on the type of mineral soil). e) Porosity, f) field capacity, g) saturated hydraulic conductivity, h) heat conductivity of (completely) dry soils, i) heat conductivity of fully saturated soils, j) heat capacity of (completely) dry soils, k) heat capacity of fully saturated soils.

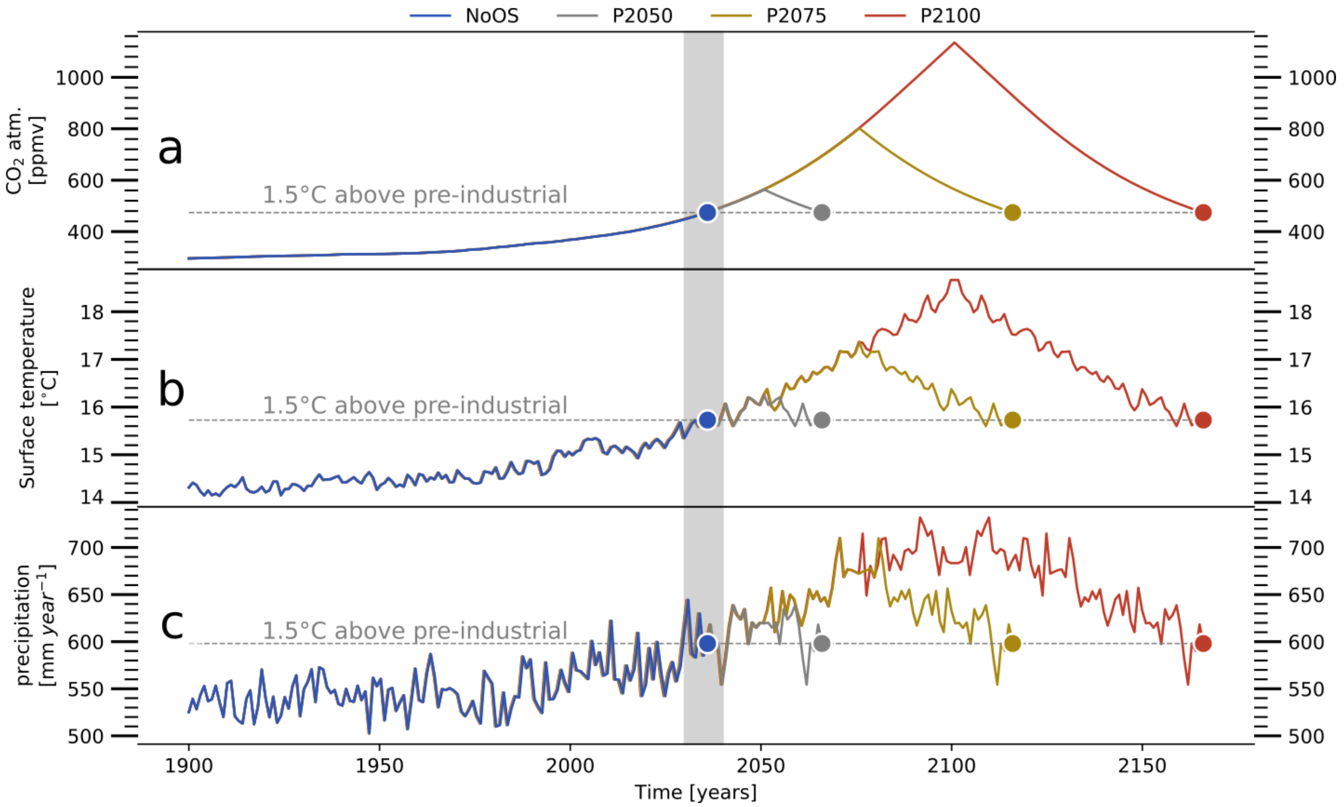

Supplementary Figure 5. Forcing

a) Atmospheric CO_2 concentration, b) (global) mean of the surface temperatures, c) and mean of the precipitation rates that were used to force the land surface model. Shaded areas indicate the period from 2030 - 2040 from which the forcing was taken for the simulations under nontransient atmospheric conditions corresponding to the target climate (PACT1.5). Note that for the investigation of the adjustment-timescales the forcing was prescribed by cycling over the years 2030 - 2040, while for the investigation of the multistability the forcing year was selected at random from the 2030 - 2040 period. Finally, it should be noted that the model is not forced by surface temperatures directly, but by atmospheric temperatures at a height of roughly 30 m and the surface incoming long- and short-wave radiative fluxes.

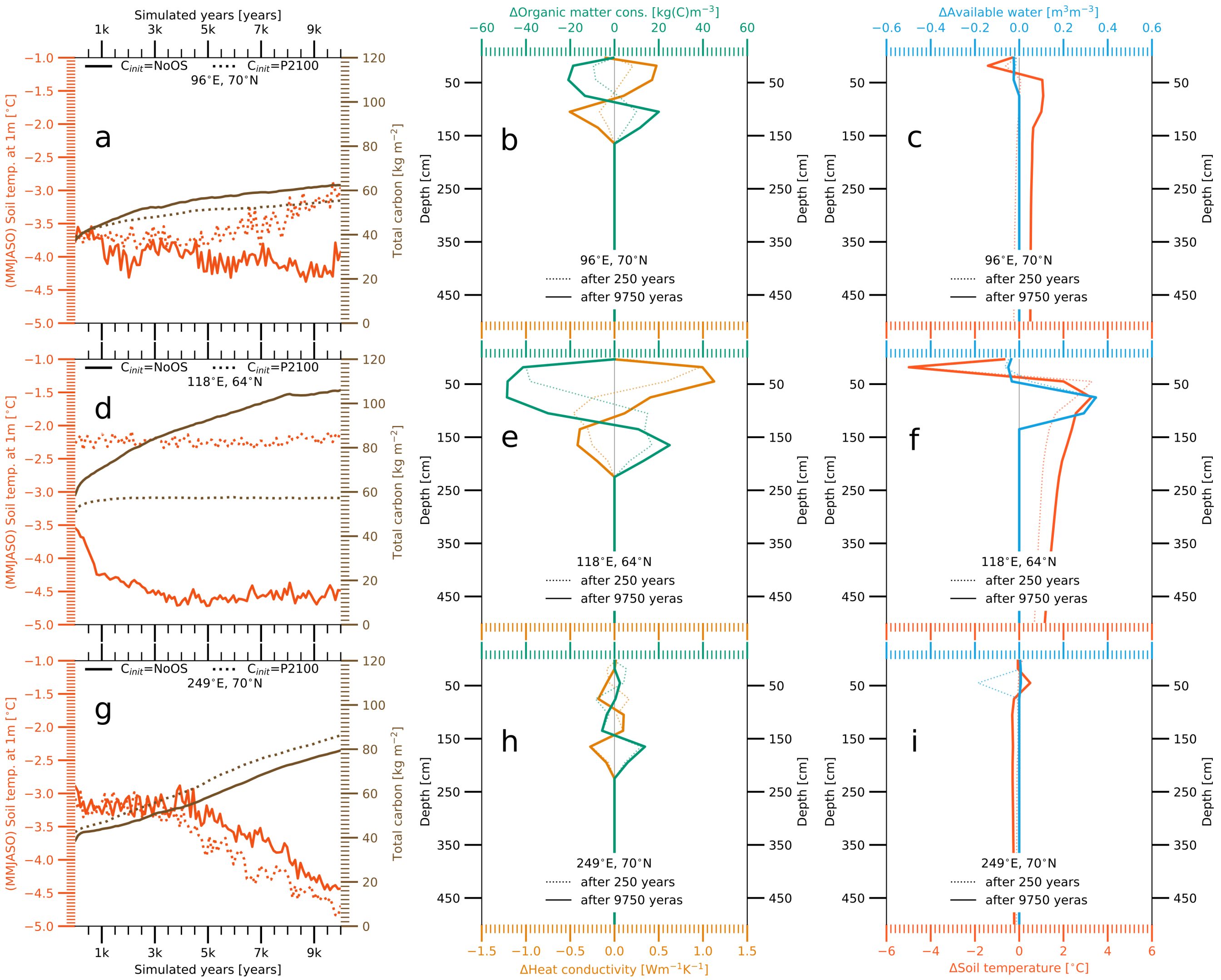

Supplementary Figure 6. Dependency of steady-state conditions on the initial soil carbon concentrations
a) Simulated May-October-mean soil temperatures at a depth of 1 m (red lines, left y-axis) and carbon densities (brown lines, right y-axis) in a grid cell at 96°E; 70°N. Solid lines refer to a simulation which was initialized with soil carbon pools before the temperature overshoot (NoOS), whereas dotted lines show a simulation that started from the pools after the overshoot (P2100). Both simulations were initialized with the same soil temperature profile and soil water content and were similarly forced with prescribed atmospheric conditions corresponding to the target climate (PACT1.5). All lines show 100-year averages. b) Difference in soil organic matter concentration (green lines, top x-axis) and heat conductivity (yellow lines, bottom x-axis) between the two simulations (P2100-NoOS), at the beginning (dotted lines) and at the end (solid lines) of a 10000-year period. c) Same as panel b but for the annual maximum (monthly-mean) soil temperature (red lines, top x-axis) and liquid water content (blue lines, bottom x-axis). d,g) Same as panel a but for grid cells located at 118°E; 64°N and 249°E; 70°N. e,h) Same as panel b but for grid cells located at 118°E; 64°N and 249°E; 70°N. f,i) Same as panel c but for grid cells located at 118°E; 64°N and 249°E; 70°N.

At 96°E; 70°N (a-c) the simulations show that the soil temperatures can be very similar for thousands of years, before diverging. This indicates that there may be thresholds with respect to the effect of soil organic matter (SOM) on the simulated soil temperatures. The simulated dynamics in the grid cell at 118°E; 64°N (d - f), are very similar to those simulated at 116°E; 62°N (see main article Fig. 4g-i). However, at 118°E; 64°N the carbon concentration appear to stabilize – almost immediately for P2100 and after about 8000 years in case of the NoOS-simulation. Finally, while in most grid-cells the carbon pools before the overshoot (OS) are larger than the post-OS pools, there are a few grid cells where the post-peak SOM concentrations are larger (g - i). However, it is not clear whether the differences between the simulations will persist or whether the carbon pools will eventually converge to the same SOM concentrations.

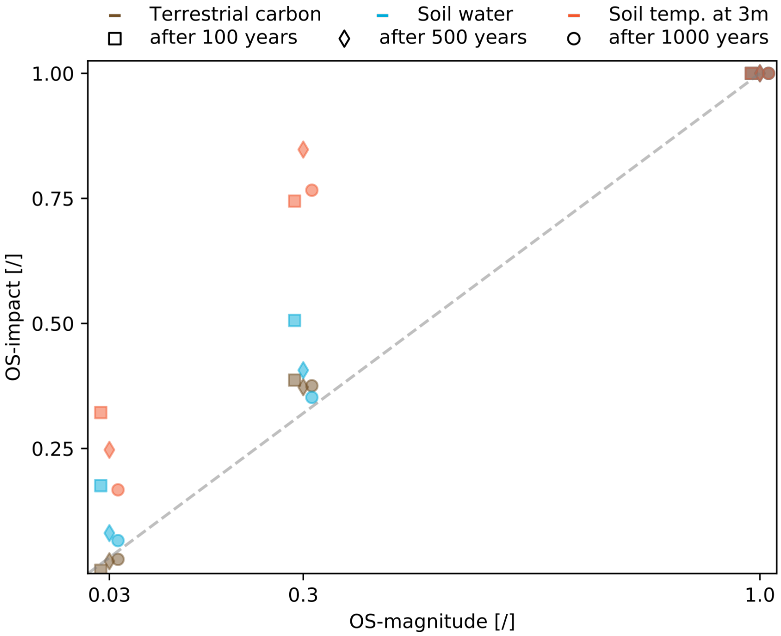

Supplementary Figure 7. Magnitude and impact of different temperature overshoots
For three time slices (after 100 (squares), 500 (diamonds) and 1000 (circles) years under nontransient atmospheric conditions), the figure shows the impact of different temperature overshoots (OS) on the total soil carbon (brown markers), water content (blue markers) and temperatures (red markers) as a function of the OSs' magnitude ($\mathrm{OSM}^{\text{P-Year}}$) – given by the overshoot-degree-years (see below). Here, we define the OS's impact ($\mathrm{OSI}_X^{\text{P-Year}}$) on a given variable ($X$) as the difference between the OS-simulation (P-Year) and the simulation without OS (NoOS):

$$\mathrm{OSI}_{\mathrm{X}}^{\text{P-Year}} = X^{\text{P-Year}} - X^{\text{NoOS}}$$

and for better comparability, we show the OS's magnitude and impact relative to the largest OS investigated in this study (P2100):

$$\mathrm{OSM}_{\mathrm{Rel}}^{\text{P-Year}} = \mathrm{OSM}^{\text{P-Year}}/\mathrm{OSM}^{\text{P2100}};\ \mathrm{OSI}_{\mathrm{Rel,X}}^{\text{P-Year}} = \mathrm{OSI}_{\mathrm{X}}^{\text{P-Year}}/\mathrm{OSI}_{\mathrm{X}}^{\text{P2100}}.$$

The comparison of the different OSs does not show any thresholds with respect to their impacts on soil carbon, -water content or -temperature. The impact of the small overshoot – P2050 – amounts to roughly 5 % - 20 % of the large OS's impact (P2100), while its magnitude is about 95 % smaller. In case of the moderate OS – P2075 –, the impacts are roughly 50 % smaller than those due to the extreme OS, while the OS's magnitude is about 70 % smaller. Rather, than threshold-behaviour this suggest a continuous dependency of the OS's impact on its magnitude. However, it should be noted that this is only true for the permafrost-affected region as a whole. An analysis of individual grid-boxes showed that there can be a substantial impact for a small OS and no further increase in the impact's strength for larger OSs, while in other grid-boxes the opposite is true (not shown). Thus, the continuous dependency arises partly from the spatial aggregation – in the more southern permafrost regions substantial impacts can already result from a smaller OS while in the more northern regions it takes a larger OS to triggered a notable impact.

Overshoot-Degree-Years: Responses in terrestrial ecosystems are often proportional to the magnitude of the causative factors in combination with the duration of the exposure to these factors. These are best characterised using cumulative measures – a prominent example being the growing degree days that describe a plant's potential heat accumulation during the growing-period. Analogously to the growing degree days, we estimate the magnitude of an OS by the overshoot-degree-years (ODY), presuming that most (slow) responses in the Arctic – such as vegetation shifts, permafrost degradation or the decomposition of soil organic matter – depend on the temperature excess as well as on the duration of the OS. With respect to the target climate (PACT1.5) we define the ODY as the summed global annual mean temperature anomaly relative to 1.5°C above pre-industrial levels. Here, it should be noted that ODY are not an established measure and it is clear that they only hold for a certain time- and temperature range, and only in the case that temperatures are well correlated with other important variables such as precipitation rates and the atmospheric GHG concentrations. For the present analyses, ODY appear to constitute a suitable measure as GHG concentrations, temperatures and precipitation rates in the Arctic are well correlated with global mean temperatures[15], and our results indicate that the lengths of the investigated OS fall into the adjustment-timescale of most key variables.

Supplementary References

1. Brown, R. D. & Brasnett, B. Canadian meteorological centre (cmc) daily snow depth analysis data, version 1., cmc_swe_mly_clim_1998to2012_v01.2. NASA National Snow and Ice Data Center Distributed Active Archive Center, Boulder, Colorado USA (2010). URL https://nsidc.org/data/NSIDC-0447/versions/1. Accessed: 06.03.2021.
2. Ekici, A. *et al.* Simulating high-latitude permafrost regions by the jsbach terrestrial ecosystem model. *Geosci. Model Dev.* **7**, 631–647 (2014).
3. Ekici, A. *et al.* Site-level model intercomparison of high latitude and high altitude soil thermal dynamics in tundra and barren landscapes. *The Cryosphere* **9**, 1343–1361 (2015). URL https://doi.org/10.5194/tc-9-1343-2015.
4. Beusch, L., Gudmundsson, L. & Seneviratne, S. I. Crossbreeding CMIP6 earth system models with an emulator for regionally optimized land temperature projections. *Geophysical Research Letters* **47** (2020). URL https://doi.org/10.1029/2019gl086812.
5. Christiansen, H. H. *et al.* The thermal state of permafrost in the nordic area during the international polar year 2007-2009. *Permafrost and Periglacial Processes* **21**, 156–181 (2010). URL https://doi.org/10.1002/ppp.687.
6. Romanovsky, V. E., Smith, S. L. & Christiansen, H. H. Permafrost thermal state in the polar northern hemisphere during the international polar year 2007-2009: a synthesis. *Permafrost and Periglacial Processes* **21**, 106–116 (2010). URL https://doi.org/10.1002/ppp.689.
7. Smith, S. *et al.* Thermal state of permafrost in north america: a contribution to the international polar year. *Permafrost and Periglacial Processes* **21**, 117–135 (2010). URL https://doi.org/10.1002/ppp.690.
8. Burke, E. J., Zhang, Y. & JKrinner, G. Evaluating permafrost physics in the coupled model intercomparison project 6 (cmip6) models and their sensitivity to climate change. *The Cryosphere* **14**, 3155–3174 (2020). URL https://doi.org/10.5194/tc-14-3155-2020.
9. Beer, C., Porada, P., Ekici, A. & Brakebusch, M. Effects of short-term variability of meteorological variables on soil temperature in permafrost regions. *The Cryosphere* **12**, 741–757 (2018). URL https://doi.org/10.5194/tc-12-741-2018.
10. Beer, C., Zimov, N., Olofsson, J., Porada, P. & Zimov, S. Protection of permafrost soils from thawing by increasing herbivore density. *Scientific Reports* **10** (2020). URL https://doi.org/10.1038/s41598-020-60938-y.
11. Porada, P., Ekici, A. & Beer, C. Effects of bryophyte and lichen cover on permafrost soil temperature at large scale. *The Cryosphere* **10**, 2291–2315 (2016). URL https://doi.org/10.5194/tc-10-2291-2016.
12. Beer, C., Fedorov, A. N. & Torgovkin, Y. Permafrost temperature and active-layer thickness of yakutia with 0.5-degree spatial resolution for model evaluation. *Earth System Science Data* **5**, 305–310 (2013). URL https://doi.org/10.5194/essd-5-305-2013.
13. Brown, J., Hinkel, K. M. & Nelson, F. E. The circumpolar active layer monitoring (calm) program: Research designs and initial results1. *Polar Geography* **24**, 166–258 (2000). URL https://doi.org/10.1080/10889370009377698.
14. Chadburn, S. E. *et al.* Carbon stocks and fluxes in the high latitudes: using site-level data to evaluate earth system models. *Biogeosciences* **14**, 5143–5169 (2017). URL https://doi.org/10.5194/bg-14-5143-2017.
15. de Vrese, P., Stacke, T., Kleinen, T. & Brovkin, V. Diverging responses of high-latitude co2 and ch4 emissions in idealized climate change scenarios. *The Cryosphere* **15**, 1097–1130 (2021). URL https://doi.org/10.5194/tc-15-1097-2021.
